# Accelerating inflammatory resolution in humans to improve endothelial function and vascular health: Targeting the non-canonical pathway for NO

**DOI:** 10.1016/j.redox.2025.103592

**Published:** 2025-03-28

**Authors:** Clement Lau, Christopher P. Primus, Asad Shabbir, Ismita Chhetri, Mutsumi Ono, Michael Masucci, Muhammad Aadil Bin Noorany Aubdool, Julie Amarin, Alexander JP. Hamers, Zara Khan, Nitin Ajit Kumar, Shanik A. Montalvo Moreira, Gani Nuredini, Miski Osman, Charlotte Whitear, Tom Godec, Vikas Kapil, Gianmichele Massimo, Rayomand S. Khambata, Krishnaraj S. Rathod, Amrita Ahluwalia

**Affiliations:** a Barts and the London Faculty of Medicine and Dentistry, Queen Mary University of London, London, UK; b Department of Cardiology, Barts Heart Centre, 2 St. Bartholomew's Hospital, Barts Health NHS Trust, London, UK; c Cardiovascular Clinical Trials Unit, Barts and the London Faculty of Medicine and Dentistry, Queen Mary University of London, London, UK

**Keywords:** Blister, Endothelium, Inorganic, Inflammation, Nitrate, Typhoid

## Abstract

**Background:**

Chronic cardiovascular diseases (CVD) are characterised by low-grade systemic inflammation in part due to reduced nitric oxide (NO) bioavailability associated with endothelial dysfunction. Bioavailability of NO can be enhanced by activation of the non-canonical pathway, through increased dietary inorganic nitrate consumption with the potential to attenuate inflammation.

**Methods:**

We sought to determine whether dietary inorganic nitrate influences the inflammatory response in models of localised (cantharidin-induced blisters) and systemic inflammation (typhoid vaccine), in healthy male volunteers and conducted two clinical trials; Blister-NITRATE and Typhoid-NITRATE respectively.

**Results:**

We show that dietary nitrate attenuates endothelial dysfunction following typhoid vaccine administration and accelerates resolution of cantharidin-induced blisters. Both phenomena were associated with an increased level of pro-resolving mediators consequent to a reduction in the expression and activity of pro-inflammatory monocytes. Moreover, we show that leukocytes of the monocyte lineage express the nitrite reductase XOR, that may drive localised nitrite reduction to elevate NO (and cGMP) to drive the protective phenotype.

**Conclusions:**

Inorganic nitrate improves endothelial function in the setting of systemic inflammation. Whilst the immediate inflammatory response appeared unaffected by inorganic nitrate treatment, during the resolution phase of the acute inflammatory response lower levels of pro-inflammatory classical inflammatory and intermediate monocytes and attenuated levels of inflammatory cytokines and chemokines were evident. We propose that this reflects a pro-resolution phenotype that may be of potential therapeutic benefit in patients with established CVD.

**Clinical trial registration:**

URL: https://www.clinicaltrials.gov; unique identifiers NCT02715635, NCT03183830.

**Table:** Non-standard abbreviations and acronyms

ANOVA	Analysis of variance
AIx	Augmentation Index
BP	Blood pressure
CAD	Coronary artery disease
CD	Cluster of differentiation
CVD	Cardiovascular disease
CXCL	Chemokine (C-X-C motif) ligand
EDTA	Ethylenediaminetetraacetic acid
ELISA	Enzyme-linked immunosorbent assay
FMD	Flow mediated dilatation
GTN	Glyceryl trinitrate
IBMX	Isobutylmethylxanthine
IL	Interleukin
IM	Intramuscular
NHS	National Health Service
NO	Nitric oxide
PWA	Pulse wave analysis
PWV	Pulse wave velocity
RCF	Relative centrifugal force
ROI	Region of Interest
SD	Standard deviation
SEM	Standard error of the mean
SST	Serum separating tubes
TNF	Tumour necrosis factor
XOR	Xanthine oxidoreductase

## 1 Introduction

It is accepted that inflammation drives the development of endothelial dysfunction and thus atherosclerosis [1]. Indeed, a multitude of circulating (neutrophils, monocytes, T-cells) and tissue-localised (resident macrophages, mast cells) immune cell types [[2], [3], [4]] have been implicated in this process. This underlies a focus on identification and development of anti-inflammatory pharmacotherapies for endothelial dysfunction in cardiovascular disease (CVD) [[4], [5], [6]]. Indeed, clinical trials testing interventions that suppress inflammation have shown efficacy in reducing major adverse cardiovascular events however, this benefit comes at a cost [7,8]. Monoclonal antibodies opposing pro-inflammatory cytokines (canakinumab, monoclonal antibody to interleukin-1β [9]) or colchicine (suppressing microtubule formation and inflammasome activation) [10,11], whilst reducing CV events (CV mortality and unplanned revascularisation for unstable angina), also caused an increased risk of infection and other side effects, thus precluding their use currently in clinical practice for chronic CVD (e.g. athercosclerosis). Thus, the strategy of repressing inflammation to reduce endothelial dysfunction has not yet led to efficacious approaches that have translated to the clinic.

More recently, it has been proposed that rather than inhibiting inflammation that accelerating the resolution of inflammation may provide safer therapeutics for chronic CVD [12], but with less risk of immune compromise and host infection. Whether this might be an option for reduction/prevention of endothelial dysfunction is unknown.

Amongst its many beneficial properties, endothelial nitric oxide (NO) exerts cardio- and vasculo-protective effects via its anti-inflammatory actions [13,14]; a key aspect of this being to protect against endothelial dysfunction [15,16]. Suppression of the expression and activity of several adhesion molecules, expressed upon specific leukocyte subsets (e.g. CD11b [17,18], PSGL1 [19]) and the endothelium (e.g. P-Selectin [20], ICAM-1 [21]), has been proposed to underlie many of the positive effects of NO upon inflammatory responses [13,22,23]. Evidence also supports a role for endothelial NO in suppression of pro-inflammatory cytokine and chemokine expression, including interleukin-1 (IL-1), IL-6, Tumour necrosis factor α (TNFα), chemokine (C-X-C motif) ligand 1 (CXCL1), CXCL2 and CCL2 (C–C motif) [[24], [25], [26]]. Separately there is also pre-clinical data suggesting that NO supports the clearing of an inflammatory response (i.e. resolution) through triggering anti-inflammatory IL-10 [27] generation from monocyte/macrophages, thus reducing neutrophil recruitment, and in turn improving endothelial function through upregulating eNOS expression and activity [24,28,29].

In CVD NO bioavailability is reduced, and thus the beneficial anti-inflammatory tonic influence, described above, over the vasculature diminished. Replacement of this reduced NO to restore the beneficial profile of endothelium-derived NO has been a much sought after goal. The organic nitrates, such as GTN, as NO donors, have not been useful in this context due to the rapid tachyphylaxis of activity as well as due to their inherent capacity to induce endothelial dysfunction *per se* [30]. Alternative strategies to deliver NO have therefore been sought. Much evidence now supports the view that activation of the non-canonical pathway for NO delivery with dietary inorganic nitrate (NO_3_^−^) offers therapeutic potential through NO delivery [31], without the issues of tolerance and tachyphylaxis associated with organic nitrates. Indeed, several studies have shown the positive effects of inorganic nitrate interventions upon vascular function. This includes our work demonstrating that it prevents ischaemia-induced endothelial dysfunction in healthy volunteers [15,16], and improves the FMD response in patients with hypertension [32] and hypercholesterolaemia [33]. These observations have been confirmed by others and in meta-analyses [34]. However, whether the beneficial effects might relate to an impact upon the immune responses key to driving endothelial dysfunction is uncertain.

To address these uncertainties, we have undertaken two studies in healthy volunteers, to allow us to test the hypothesis that dietary inorganic nitrate alters inflammatory responses and thus prevents systemic inflammation-induced endothelial dysfunction. The Typhoid-NITRATE study (NCT02715635) assessed the impact of inorganic nitrate treatment upon systemic inflammation-induced endothelial dysfunction. The Blister-NITRATE study (NCT03183830) assessed the impact of inorganic nitrate treatment upon localised inflammation in the skin. We elected to study responses only in males since previous evidence shows that women resolve blister inflammation too fast to study practically. Additionally, healthy women do not experience systemic-inflammation induced endothelial dysfunction in response to typhoid vaccine [35]. Thus, in these studies the impact of dietary nitrate was studied in men only.

## 2 Methods

***Clinical trial approvals and declarations****.* The Typhoid-NITRATE (REC reference No. 15/LO/0789) and Blister-NITRATE (REC reference No: 16/LO/0160) studies were approved by NRES Committee London – City Road & Hampstead and The London – City & East Research Ethics Committee respectively. Both studies are registered at Clinicaltrials.gov (NCT02715635 and NCT03183830). For mechanistic studies on human blood ethical approvals were gained from the Queen Mary Ethics of Research Committee (QMERC2018/69). All studies were performed according to the ethical principles for medical research involving human subjects or samples, and the Declaration of Helsinki.

***Volunteers***. Healthy volunteers were recruited if they offered informed consent, were aged 18–45 years, with normal resting blood pressure (<140/90 mmHg). Exclusion criteria included: history of serious illness or recent infections or trauma, use of any systemic medication, use of mouthwash or tongue scraping (since the oral microbiome is implicated in a functional non-canonical pathway for NO generation [31]), recent (within 3 months) or current antibiotic use, history or recent treatment (within 3 months) for any oral condition including gingivitis,periodontitis or halitosis, typhoid vaccine in the previous 6 months, history of a blood-borne infection such as hepatitis B virus, hepatitis C virus or HIV. Healthy women were excluded from these studies since our previous work has demonstrated that women do not suffer systemic inflammation induced endothelial dysfunction in response to typhoid vaccine, and resolve cantharidin-induced inflammation much faster (than men); meaning that there are no blisters at the 72h timepoint.

All clinical study activities were performed in a quiet and temperature controlled (22–24 °C) laboratory. Volunteers were fasted for 3h prior to each study visit. Participants refrained from caffeine or strenuous activity and adhered to a low nitrate diet for 24h prior to each study visit. Volunteers were recruited, consented and then followed the protocol for either Typhoid-NITRATE or Blister-NITRATE (see Fig. S1 for protocol schematics).

***Intervention and placebo***. After screening, volunteers were randomly assigned in a 1:1 fashion to receive either 140 mL (∼800 mg) of nitrate-rich beetroot juice or placebo nitrate-deplete beetroot juice (James White Drinks Ltd., Beet It® Sport Nitrate 400). The placebo juice was manufactured using an anion exchange resin as previously described [36]. There were no visual, smell or taste differences between nitrate-rich or nitrate-deplete juice.

***Typhoid-vaccine induced endothelial dysfunction study (Typhoid-NITRATE)***. For this study an established and validated protocol was employed [37,38]. *Part I*. 62 healthy male volunteers were consented. On day 1, volunteers attended the clinic at 3pm for baseline investigations. Volunteers underwent blood pressure (BP), pulse wave analysis (PWA) and pulse wave velocity (PWV) measurement. Blood, urine and saliva samples were collected for clinical haematology, biochemistry and ozone chemiluminescence assessment. An additional blood sample was drawn into a citrate containing BD Vacutainer® (Becton, Dickinson and Company, USA) for inflammatory cell flow cytometry analysis. FMD was measured. Volunteers were randomised to either treatment arm. Volunteers consumed juice for a total of 7 days starting from day 2. Volunteers attended on day 7 at 8am to receive typhoid vaccine. and then returned at 3pm on the same day to undergo repeat investigations as performed on day 1. *Part II*. 16 participants were recruited. On day 1, volunteers attended the clinic at 3pm for baseline investigations. Volunteers underwent BP measurement and blood, urine and saliva samples were collected for clinical haematology, biochemistry and ozone chemiluminescence. FMD was measured according to gold standard protocols [39]. Following acquisition of FMD responses GTN-induced brachial artery dilatation was also measured. This GTN measurement was conducted once baseline diameter had returned to pre-FMD levels and at least 10 min following FMD assessment as per guidelines [39]. Following these measurements volunteers were randomly assigned to either treatment arm and then returned 7 days later at 8am to receive typhoid vaccine. At 3pm on this day volunteers returned to undergo repeat investigations as per day 1. On day 8, participants returned at 3pm for the final repeat of all assessments as per day 1. The visit scheme is summarised in Fig. S1A. For vaccine administration 0.5 mL of a typhoid vaccine (Sanofi Pasteur Ltd., TYPHIM Vi®) containing 25 μg of purified Vi capsular polysaccharide Salmonella typhi (Ty2 strain) was administered as an intramuscular (IM) injection into the deltoid muscle.

***Cantharidin-induced blister study (Blister-NITRATE)***. For this study an established and validated protocol was employed [[40], [41], [42]]. A total of 36 healthy volunteers were consented. The intervention was ingested from the final visit of week 1 (control week) until the end of week 2 (treatment week), totalling 8 days. The visits for each week were identical (Fig. S1B). At visit 1 (day 1 and day 8), volunteers measured their own BP at 15-min intervals in triplicate for 60 min, using a portable BP machine (Omron 715IT, Kyoto, Japan), with the second and third readings used for analysis. Participants provided samples of plasma, urine and saliva for processing. Following this, 10 μL of cantharidin (Cantharone 0.1 %, Dormer Laboratories, Toronto, Canada) was mixed with 60 μL of clinical grade acetone, and 10 μL of this solution applied to the ventral aspect of the forearm on filter-paper discs and dressed. At visit 2, another blister was created with a further application of cantharidin. At visit 3, BP and clinical samples were taken as described. Blister fluid exudates were harvested at 24-h (acute phase) and 72-h (resolution phase) by carefully piercing the side of the blister with a 25-gauge needle and rolling a pipette tip over the blister surface to express fluid. This exudate was collected using a pipette tip, and the exudate stored in a pre-weighed Eppendorf on ice. Blister exudate was weighed to assess the volume harvested per blister. Total cell count was calculated for whole blood and blister exudate using Tüerk's solution and cells were counted using a haemocytometer. Blister exudate was then centrifuged at 400*g* for 5 min at 4 °C. The supernatant was snap frozen and stored at −80 °C until biochemical analysis for cytokine/chemokine levels.

*BP****measurement***. BP was measured in triplicate, with the cuff placed on the non-dominant arm in accordance with established guidance [43].

***PWV and PWA measurements***. PWV and central BP were measured in 62 volunteers (31 in each group) using a Vicorder® device (Skidmore Medical Ltd.) detector as per validated procedures [44].

***Measurement of brachial artery diameter***. Brachial artery ultrasound was used to measure FMD and GTN-induced brachial artery dilatation in a sub-cohort of 16 volunteers (8 in each group) as per gold-standard guidelines [45,46].

***Blood, urine and saliva sampling***. Whole blood was obtained from a vein in the antecubital fossa using a 21-gague butterfly needle and collected directly into BD Vacutainer® EDTA, SST and citrate containing blood tubes. Clean catch mid-stream urine samples were collected in sterile gallipots, aliquoted into 1.5 mL microcentrifuge tubes for nitrite and nitrate quantification. Participants were instructed to allow saliva to drip from their tongue/lower lip into a sterile gallipot. This saliva was aliquoted and spun at 1300*g* for 10 min at 4 °C, and supernatant snap frozen and stored at −80 °C for various biochemical analyses (including nitrate, nitrate, cGMP and cytokines/chemokines.

***Inflammatory cell flow cytometry***. Flow cytometry was used to assess systemic inflammatory response and blister fluid infiltrate using a BD LSRFortessa™ Flow Cytometer, and data recorded using BD FACSDiva™ analysis software as per standard procedures and fully described in the supplementary information.

***Leukocyte XOR expression***. To determine whether leukocytes might be the site of nitrite reduction, by XOR, expression levels of the enzyme were measured. Blood was collected and mixed 1:1 v/v with PBS. Peripheral blood mononuclear cells (PBMCs) and polymorphonuclear cells (PMNs) were isolated using density centrifugation and anti-human xanthine oxidase (1:1000; Abcam Cat# ab133268, RRID:AB_11154903), binding to cells analysed as above using flow cytometry. For representative images of leukocytes images were acquired using Amnis® Imagestream X MK2 and analysed using Ideas software v6.2. Representative histogram images were produced using FlowJo v10.8.1.

***Immunoblotting.*** Human PBMC and PMN homogenates were subjected to SDS/PAGE (0.1 % w/v) immunoblotting analysis using an anti-human XOR rabbit antibody (1:2000, Abcam 133268 RRID:AB_11154903).

***qPCR assessment of leukocyte XOR expression***. RNA from PBMCs and PMNs was extracted, using Nucleospin RNA extraction kit (Macherey Nagel, Germany), according to manufacturer guidelines. cDNA was synthesised and qPCR analysis carried out with SYBR green (ThermoFisher Scientific, UK), using specifically designed primers for hXDH 5′-CTCAGTCAGCCTCTCGCCAT-3′ and 5′-TATCCACGTCACACGCTCCC-3’. qRT-PCR was performed using an ABI Quantstudio 7 sequence detection system.

***Inflammatory mediator analysis****.* Cytokine and chemokine expression profiles in acute and resolution phase blisters following cantharidin application and in plasma collected from volunteers before and 8h following typhoid vaccine administration were analysed. All analysis was conducted blind to treatment allocation by an external commercial organisation (LaboSpace) using a bead array (IDEXX ProCyte Dx Haematology Analyser).

Soluble CD62L was quantified using a sandwich enzyme linked immunosorbent assay (ELISA) (human L-selectin/CD62L, R&D Systems, catalogue DY728) and hsCRP using a sandwich ELISA (Human CRP ELISA Kit, catalogue KHA0031) as per the manufacturer's instructions.

As an indirect measure of apoptosis in blisters the levels of lactate dehydrogenase (LDH) activity in the cell free blister fluid supernatant were assessed as per the manufacturer's instructions (CyQuant LDH cytoxicity assay kit, C20300, Invitrogen, UK). For determination of lactate levels the l-Lactate assay kit (ab65331, Abcam, UK) was used as per manufacturer's instructions.

***Assessment of nitrite, nitrate and cGMP levels and leukocyte nitrite reductase activity***. To assess nitrite (NO_2_^−^) and nitrate (NO_3_^−^) levels within plasma, saliva and urine [33,47] an ozone-based chemiluminescence method was used that captures NO following chemical reduction of both anions. For all plasma assessments we subtracted the average estimated contamination levels of nitrate and nitrite occurring within 17 commercially-purchased blood collection tubes. To each tube 4 mL of nitrate/nitrite-free distilled water was added and this tube subjected to identical processing to that of blood samples. The level of nitrate was 0.64 ± 0.2 μM and nitrite was 0.41 ± 0.03 μM. The chemiluminescent method was also used to measure nitrite-reductase activity [48] as described previously. cGMP concentration in plasma samples were quantified using an enzyme immunoassay Biotrak kit (GE Healthcare, catalogue RPN226) as per manufacturer's instructions.

***A priori sample size estimation, blinding and statistical analysis*.** For Typhoid-NITRATE Part I, it was expected that the typhoid vaccine would cause a reduction in FMD of 1.5 % [37]. This, with a SD of 1.1 and a power of 80 % resulted in 31 in each group being required for statistical power. A further 16 additional volunteers were recruited in Part II for assessment of GTN-induced brachial artery dilatation [49]. For Blister-NITRATE,

For the primary outcome of emigrated neutrophils, our previous studies assesseing sex differences demonstrated a difference of 50 % in cell count (mean 0.6x10^6^ cells/mm^3^, SD = 0.2x10^6^) [50], and thus with a power of 90 % 12 subjects are required per group assuming that dietary nitrate will reduce counts to a similar level. To account for technical failures (participant-induced accidental blister perforation) or potential dropout, the total volunteer recruitment was increased to 35 due to accidental burst blisters. This also gives >90 % power to detect a rise in nitrite as per previous observations [16].

Data are show as mean ± standard error of mean (SEM), unless otherwise stated. For the typhoid study 2-way ANOVA was used for comparisons over time between groups followed by post hoc Dunnett's multiple comparisons test for comparisons to baseline. For the blister study a Student's paired *t*-test was used for within group comparisons. Comparison between groups was determined by comparing the change from baseline between groups using a Student's unpaired *t*-test. A Fisher's Exact test for contingency analysis of resolution of blisters was conducted. Statistical analysis was performed using GraphPad Prism version 8.

## 3 Results

**Fig. 1:**
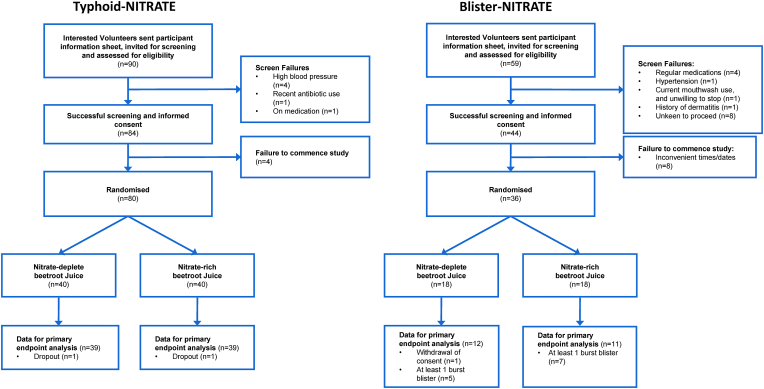
Consort flow charts for (A) Typhoid-NITRATE and (B)Blister-NITRATE. BP, blood pressure; FMD, flow-mediated dilatation; GTN, glyceryl trinitrate.

Baseline demographics for Typhoid-NITRATE and Blister-NITRATE are shown in Table 1. Intervention and placebo arms of both studies were matched. A total of 78 healthy volunteers were recruited into Typhoid-NITRATE, with 62 into *Part I*, and 16 into *Part II*. Due to accidental blister rupture the total of recruited healthy volunteers into Blister-NITRATE was increased to 36 to provide sufficient intact blisters for analysis as per *a priori* sample size estimations (i.e. n = 12 matched samples at 24 and 72h). See Fig. 1 for Consort flow diagrams.

**Table 1:** Baseline demographics of volunteers recruited into the Typhoid-NITRATE and Blister-NITRATE studies. Data are shown as mean ± SEM.

*Typhoid-NITRATE* *Part I and Part II*	Placebo	Dietary Nitrate	*Blister-NITRATE*	Placebo	Dietary Nitrate
Volunteers (n)	39	39	Volunteers (n)	17	18
Age (yr)	26.7 ± 1.0	25.6 ± 0.8	Age (yr)	28.2 ± 7.1	29.1 ± 6.4
Weight (kg)	74.8 ± 2.4	76.2 ± 2.0	Weight (kg)	77.1 ± 3.0	76.6 ± 1.7
Height (m)	1.8 ± 0.01	1.8 ± 0.01	Height (m)	1.8 ± 0.01	1.8 ± 0.01
BMI (kg/m^2^)	23.9 ± 0.5	23.7 ± 0.4	BMI (kg/m^2^)	24.1 ± 3.4	23.6 ± 1.9
Baseline SBP (mmHg	119.4 ± 1.5	118.7 ± 1.6	Baseline SBP (mmHg)	115.0 ± 6.5	117.0 ± 8.8
Baseline DBP (mmHg)	67.3 ± 1.1	67.1 ± 1.4	Baseline DBP (mmHg)	68.0 ± 6.5	68 ± 7.7
Heart rate (bpm)	68.2 ± 1.9	67.0 ± 1.6	Heart rate (bpm)	67.3 ± 2.1	67.2 ± 3.1
Leucocyte count (x10^9^/L)	6.3 ± 0.2	6.1 ± 0.2	Leucocyte count (x10^9^/L)	5.3 ± 1.5	6.5 ± 2.0
Creatinine (μmol/L)	84.1 ± 1.8	84.5 ± 1.7	Creatinine (μmol/L)	85.0 ± 10.3	83 ± 8.2
Urate (μmol/L)	326.8 ± 10.5	328.1 ± 11.7	Urate (μmol/L)	363 ± 67.7	310 ± 43.4
Methaemoglobin (%)	0.7 ± 0.1	0.7 ± 0.1	Methaemoglobin (%)	0.4 ± 0.3	0.4 ± 0.3

***Inorganic nitrate increases plasma, urine and saliva nitrate and nitrite concentrations***. As previously demonstrated, dietary inorganic nitrate ingestion augmented both nitrate and nitrite concentrations in plasma, saliva and urine (Table 2 and Table S1), whilst the placebo had no effect upon anion levels. In the Typhoid-NITRATE study, the change from baseline at the 8h timepoint of plasma nitrite concentration in the placebo arm was 0.05 ± 0.08 μM (n = 38), whilst in the dietary nitrate-treated arm it was 0.53 ± 0.18 μM (n = 39) (P = 0.016). In the Blister-NITRATE study the change from baseline in nitrite concentration was 0.10 ± 0.09 μM (n = 17) for the placebo arm and 0.57 ± 0.20 μM (n = 18) in the dietary nitrate arm (P = 0.046) (Table 2 and Table S1). For these studies we elected to aim for a dose of approximately 8–12 mmol of nitrate/day as per previous studies conducted by our laboratory in healthy volunteers [51]. Analysis of juice samples from both Typhoid-NITRATE and Blister-NITRATE studies demonstrated a mean concentration of nitrate in the nitrate-rich beetroot juice of 106.9 ± 4.6 mM versus 1.3 ± 0.3 mM in the placebo arm (*P* < 0.0001, unpaired *t*-test), equivalent to an effective dose of 14.9 ± 0.6 mmol (n = 47) vs 0.06 ± 0.01 mmol (n = 46). The rise in circulating nitrite levels was associated with statistically significant increases in circulating levels of cGMP (the NO effector molecule) i.e. change from baseline at 8h following Typhoid vaccine of −1.6 ± 0.6 nM in the placebo treated volunteers and 0.2 ± 0.4 nM (*P* = 0.02) in the dietary nitrate treated volunteers measured in n = 22 available samples for each group i.e. 44 volunteers in total.

**Table 2:** **Dietary nitrate elevates levels of nitrate and nitrite, measured by ozone chemiluminescence, in plasma, urine and saliva**. Data are shown as mean ± SEM, with statistical significance shown as P values for within group comparisons of paired Students T test. Missing samples due to technical failure. Data shown represents baseline and a single timepoint post-intervention for Typhoid-NITRATE, this is on day 6 and 8 h following typhoid injection. For Blister-NITRATE the measurements were conducted on samples from visit 1 for baseline and then on day 6 following initiation of the intervention (visit 3).

	Placebo				Dietary Nitrate			
***Typhoid-NITRATE*** ***Part I and Part II***	*n* value	Baseline	Post Placebo	Within group comparison (*P*)	*n* value	Baseline	Post Nitrate	Within group comparison (*P*)
Plasma [nitrate] (μM)	38	50.7 ± 6.4	62.6 ± 14.7	0.488	39	47.6 ± 5.2	352.1 ± 19.6	<0.0001
Plasma [nitrite] (μM)	38	0.4 ± 0.08	0.4 ± 0.11	0.5714	39	0.4 ± 0.1	0.9 ± 0.1	0.0047
Saliva [nitrate] (μM)	39	590.2 ± 81.5	542.0 ± 48.6	0.5037	38	701.1 ± 163.9	7611 ± 743.5	<0.0001
Saliva [nitrite] (μM)	39	203.3 ± 22.4	197.3 ± 20.3	0.7966	38	279.2 ± 34.5	1740 ± 241.1	<0.0001
Urine [nitrate] (mM)	39	1.2 ± 0.1	1.1 ± 0.1	0.269	39	1.2 ± 0.2	15.8 ± 1.6	<0.0001
Urine [nitrite] (μM)	39	0.3 ± 0.07	0.3 ± 0.03	0.7678	39	0.2 ± 0.04	1.4 ± 0.3	<0.0001

***Blister-NITRATE***	*n* value	Baseline	Post Placebo	Within group comparison (*P*)	*n* value	Baseline	Post Nitrate	Within group comparison (*P*)

Plasma [nitrate] (μM)	17	37.1 ± 1.7	32.4 ± 3.9	0.2146	18	43.7 ± 5.3	133.8 ± 28.7	0.003
Plasma [nitrite] (μM)	17	0.68 ± 0.2	0.78 ± 0.15	0.2915	18	0.63 ± 0.2	01.2 ± 0.3	0.0106
Saliva [nitrate] (μM)	16	613.3 ± 118.0	564.3 ± 97.1	0.693	18	465.9 ± 105	6875 ± 1035	<0.0001
Saliva [nitrite] (μM)	16	247.5 ± 33.0	287.9 ± 47.7	0.2961	18	307.8 ± 76.5	1535 ± 313.0	0.001
Urine [nitrate] (mM)	17	7.0 ± 1.3	7.7 ± 1.9	0.6652	18	8.0 ± 1.3	27.2 ± 6.6	0.0088
Urine [nitrite] (μM)	17	0.2 ± 0.1	0.2 ± 0.1	0.2924	18	0.3 ± 0.08	0.9 ± 0.1	0.021

Nitrate-rich beetroot juice was safe with no reported adverse reactions, no changes in basic biochemical measures or clinically significant increases in methaemoglobin levels (Table S2). In addition, this dose of dietary nitrate, whilst increasing circulating nitrate and nitrite concentrations had negligible effects upon BP or heart rate (Table S3).

**Fig. 2:**
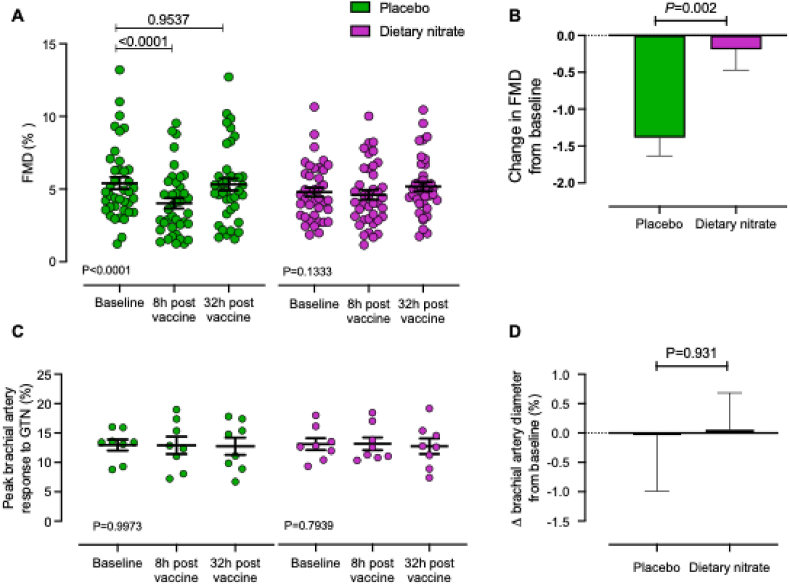
**Dietary nitrate prevents systemic inflammation-induced endothelial dysfunction.** (A) Typhoid-NITRATE *Part I:* FMD response 8 and 32 h following typhoid vaccination in placebo versus nitrate treated groups. *n* = 78. B) Comparison between the treatment groups of the change in FMD response at 8h from baseline. Typhoid-NITRATE *Part II:* (C) GTN induced brachial artery dilatation. Maximal dilatation of brachial artery in response to GTN in volunteers treated with placebo versus nitrate measured at 8- and 32-h post-vaccination *n* = 16. D) Comparison between the treatment groups of the change in GTN response at 8h from baseline.Statistical significance determined by 1-way ANOVA, with Dunnett's post hoc analysis for multiple group analysis and unpaired *t*-test for comparison of change in response at 8h compared to baseline. All data are expressed as mean ± SEM. FMD, flow-mediated dilation; GTN, glyceryl trinitrate.

***Inorganic nitrate protects against typhoid vaccine-induced endothelial dysfunction*.** As expected, typhoid vaccination was associated with an increase in circulating leukocyte numbers indicating efficacy of the vaccine, confirming previous observations [37] (Table 3). A significant reduction in FMD, indicating vascular dysfunction, was observed in participants treated with placebo juice (absolute FMD reduction of 1.4 % ± 1.6 %, *P* < 0.0001), equivalent to a 21.2 ± 6.0 % reduction of the response. In contrast, there was no change in FMD in those individuals receiving dietary nitrate. There were no differences in brachial artery responses to sublingual GTN between timepoints or between treatment groups with either treatment indicating that changes in FMD were not due to altered smooth muscle function (Fig. 2, Fig. S2). In addition, there were no differences in either the baseline diameter of the brachial artery or the shear rate following cuff deflation and restoration of flow between timepoints or between treatments (Table S4). Furthermore, we found no evidence of alterations in vascular stiffness as reflected by an absence of an impact of either typhoid vaccination or dietary intervention on PWV, PWA or augmentation index (AiX) measures (Table S3).

**Table 3:** **Typhoid vaccine elevates white cell count**. Data are shown as mean ± SEM. Statistical analysis conducted using 2-way ANOVA (mixed effects) with P value shown comparing the two treatment groups. Post-tests conducted using Sidak's post-test comparing baseline to each timepoint within group shown as ∗∗∗∗ for P < 0.0001. The n values for each measurement shown in brackets with missing values representing technical failure.

	Placebo			Dietary Nitrate			Between group comparison (*P*)
***Typhoid-NITRATE*** ***Part I and Part II***	Baseline	8 h	32 h	Baseline	8 h	32 h	
White cell count (x10^9^/l)	6.3 ± 0.2 (37)	9.9 ± 0.4∗∗∗∗ (37)	6.5 ± 0.2 (38)	6.1 ± 0.2 (39)	9.3 ± 0.3∗∗∗∗ (37)	6.5 ± 0.2 (39)	0.3618
Neutrophils (x10^9^/l)	3.5 ± 0.1 (37)	6.8 ± 0.3∗∗∗∗ (37)	3.6 ± 0.1 (38)	3.4 ± 0.2 (39)	6.5 ± 0.3∗∗∗∗ (37)	3.9 ± 0.2 (39)	0.9566
Monocytes (x10^9^/l)	0.5 ± 0.02 (37)	0.7 ± 0.03∗∗∗∗ (37)	0.5 ± 0.02 (38)	0.5 ± 0.02 (39)	0.6 ± 0.03∗∗∗∗ (37)	0.5 ± 0.02 (39)	0.0785
Lymphocytes (x10^9^/l)	2.2 ± 0.08 (37)	2.1 ± 0.1 (37)	2.1 ± 0.1 (38)	2.0 ± 0.09 (39)	1.9 ± 0.09 (37)	1.8 ± 0.08∗ (38)	0.0425

**Fig. 3:**
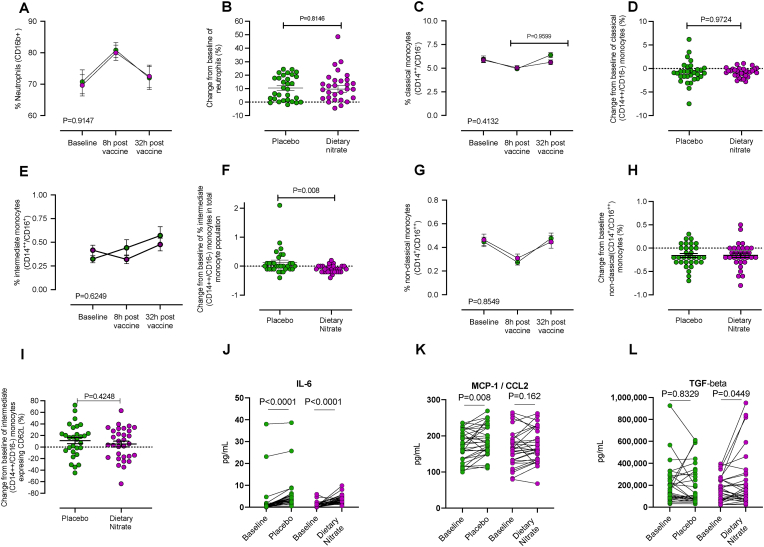
**Dietary nitrate suppresses systemic inflammation.** The proportion of leukocyte subtypes CD16b^+^ neutrophils (A,B), CD14^++^/CD16^-^ classical monocytes (C,D), CD14^++^/CD16^+^intermediate monocytes (E,F) and CD14^+^/CD16^++^ non-classical monocytes (G,H) at 8 and 32h following typhoid vaccination in placebo versus nitrate treated groups *n* = 62 and change from baseline in proportion respectively. Panel I shows the change from baseline in the proportion of CD62L^+^/CD14^++^/CD16^+^ monocytes, (I) change in % intermediate pro-inflammatory monocytes expressing CD62L at 8h from baseline. Individual paired datasets are shown with paired *t*-test for comparison of 8h to baseline. Circulating levels of pro-inflammatory IL-6 (J) and CCL2 (K) and anti-inflammatory TGFbeta (L) at baseline and at 8h following Typhoid vaccine administration in placebo following typhoid vaccination in placebo versus nitrate treated groups. Statistical significance determined by mixed effects analysis with Dunnett's post hoc analysis for within group comparison to baseline over time in Figure A,E,C,G and unpaired *t*-test for comparison of change in response at 8h compared to baseline in B,D,F,H and I. For cytokine analysis paired t-tests were conducted within groups (panels J,K,L). All data are expressed as mean ± SEM.

***Inorganic nitrate influences systemic inflammatory responses following typhoid vaccine***. Baseline leukocyte numbers were similar at baseline in the treatment groups in the Typhoid-NITRATE study and unaffected by treatment (Table 3). A transient systemic inflammatory response was observed 8h after typhoid vaccination, with increases in WCC, in both treatment groups, driven almost entirely by an increase in circulating neutrophil numbers (Table 3, Fig. 3A and B). Analysis of a subset of available samples demonstrated a small and similar, but not statistically significant, rise in hsCRP at the 8h time-point in both treatment groups (i.e. control vs placebo of 0.96 ± 0.2 (baseline) and 1.14 ± 0.2 mg/L (8h) (n = 22) and for control vs dietary nitrate of 0.93 ± 0.2 (baseline) and 1.14 ± 0.2 mg/L (n = 22)). Whilst dietary nitrate treatment did not alter the rise in WCC or neutrophil numbers at 8h (Fig. 3B), there was a statistically significant suppression (reflected as change from baseline) of the pro-inflammatory intermediate (CD14+/CD16++) monocyte subtype as well as lymphocyte numbers (Table 3, Fig. 3F). No change in either the non-classical (Fig. 3G and H) or classical monocyte numbers were observed (Fig. 3).

In participants treated with dietary nitrate there was evidence of reduced activation of intermediate monocytes reflected by decreased CD62L (Fig. 3I). No other differences in activation marker on neutrophils or monocyte subsets were observed (Figs. S3–S6). The reduction in CD62L expression on intermediate monocytes was not due to increased shedding since there were no differences in soluble CD62L levels between the groups (baseline and 8h post-typhoid plasma [CD62L] of Placebo: 1296.6 ± 47.3 and 1281.9 ± 42.5 ng/mL versus dietary nitrate group: 1266.0 ± 41.2 and 1246.1 ± 46.2 ng/mL respectively (n = 31 for each).

A multiplex bead array of plasma demonstrated that for many cytokines/chemokines concentrations were below the level of detection, however statistically significant increases in IL-6 and CCL2 were evident in the placebo group at the 8h timepoint, indicating a low-level inflammatory stimulus. Interestingly, whilst a similar rise in IL-6 occurred in those treated with dietary nitrate, the rise in CCL2 was completely absent in this group (Fig. 3J and K). In addition, whilst there were no statistically significant changes in the expression of the anti-inflammatory cytokines measured in the placebo-treated volunteers at 8h, in those treated with dietary nitrate a statistically significant rise in both TGFβ and IL-35 (Table 4, Fig. 3L) were evident.

**Table 4:** **Exploratory analysis of levels of pro- and anti-inflammatory cytokines and chemokines at baseline and at 8h following Typhoid vaccine**. Data suggests that dietary nitrate drives the cytokine/chemokine profile in low level systemic inflammation towards resolution. Data are shown as mean ± SEM of n = 31 volunteers in each group. Statistical analysis conducted using paired *t*-test for within group comparisons and unpaired *t*-test for between group comparison of change from baseline. Bold numbers show statistically significant differences.

Mediator (pg/m)	Placebo				Dietary Nitrate				
	Baseline	Post Placebo	Within group *t*-test (*P*)	Change from baseline	Baseline	Post Nitrate	Within group *t*-test (*P*)	Change from baseline	Between group comparison (P)
***Pro-inflammatory Cytokines***									
TNFα	2.7 ± 1.3	2.8 ± 1.1	0.8492	0.05 ± 0.3	1.1 ± 0.3	1.2 ± 0.3	0.4719	0.1 ± 0.2	0.8303
IL-1β	0.9 ± 0.5	0.9 ± 0.4	0.6509	−0.08 ± 0.2	0.2 ± 0.07	0.2 ± 0.06	0.5056	−0.04 ± 0.07	0.8426
IL-1α	0.7 ± 0.2	0.6 ± 0.2	0.7062	−0.08 ± 0.2	0.4 ± 0.1	0.5 ± 0.1	0.2886	0.1 ± 0.1	0.4254
IL-6	**2.7 ± 1.4**	**5.7 ± 0.2**	**<0.0001**	1.1 ± 0.2	**1.1 ± 0.2**	**3.5 ± 0.3**	**<0.0001**	3.5 ± 0.3	0.3185
IL-8 (CXCL8)	8.8 ± 1.4	9.9 ± 2.2	0.5229	1.1 ± 1.7	7.5 ± 0.7	7.3 ± 0.7	0.6121	−0.2 ± 0.4	0.4489
IFNγ	4.3 ± 2.1	3.2 ± 1.3	0.2633	−1.1 ± 0.9	5.6 ± 1.9	3.0 ± 1.1	0.025	86.3 ± 121.8	0.4762
***Pro-inflammatory Chemokines***									
CXCL1 (KC-1)	405.6 ± 61.5	388.9 ± 60.4	0.5474	−16.7 ± 27.5	365.8 ± 86.5	388.2 ± 106.7	0.3917	22.4 ± 25.8	0.3032
CXCL2 (MIP-2)	1995 ± 245.2	2123 ± 278.0	0.2146	128 ± 154.3	2268 ± 266.7	2354 ± 279.7	0.003	86.3 ± 121.8	0.8325
CXCL5 (NEA78)	3320 ± 475.2	3532 ± 541.1	0.2915	211.5 ± 300.9	3036 ± 429.7	3073 ± 475.5	0.8365	36.5 ± 175.4	0.6172
CX3CL1 (Fractalkine)	3123 ± 1695	3178 ± 1765	0.6005	54.5 ± 103.0	849.4 ± 304.3	839.6 ± 278.7	0.8586	−9.9 ± 54.8	0.5832
CCL2 (MCP-1)	**172.9 ± 7.5**	**188.4 ± 7.6**	**0.0081**	15.5 ± 5.5	163.8 ± 8.6	171.5 ± 8.3	0.1662	7.7 ± 5.4	0.313
CCL3 (MIP-1α)	28.9 ± 8.8	34.3 ± 8.6	0.2522	5.4 ± 4.6	29.5 ± 5.9	34.3 ± 6.4	0.2467	4.9 ± 4.1	0.9284
CXCL12 (SDF-1)	176.5 ± 52.2	208.7 ± 53.1	0.3728	32.2 ± 35.6	70.3 ± 27.4	86.6 ± 28.5	0.4405	16.3 ± 20.8	0.701
***Anti-inflammatory Cytokines***									
IL-10	0.13 ± 0.05	0.19 ± 0.1	0.4912	−0.08 ± 0.2	0.49 ± 0.4	0.7 ± 0.4	0.178	0.2 ± 0.1	0.3014
TGFβ	50364 ± 18574	52106 ± 17642	0.8965	50364 ± 0.04580	**36322 ± 11439**	**70276 ± 25034**	**0.0367**	50364 ± 0.05	0.1201
IL-35	200555 ± 33859	194560 ± 31895	0.8329	474.4 ± 28913	**163014 ± 20269**	**231498 ± 44645**	**0.0449**	68484 ± 32712	0.1245

**Fig. 4:**
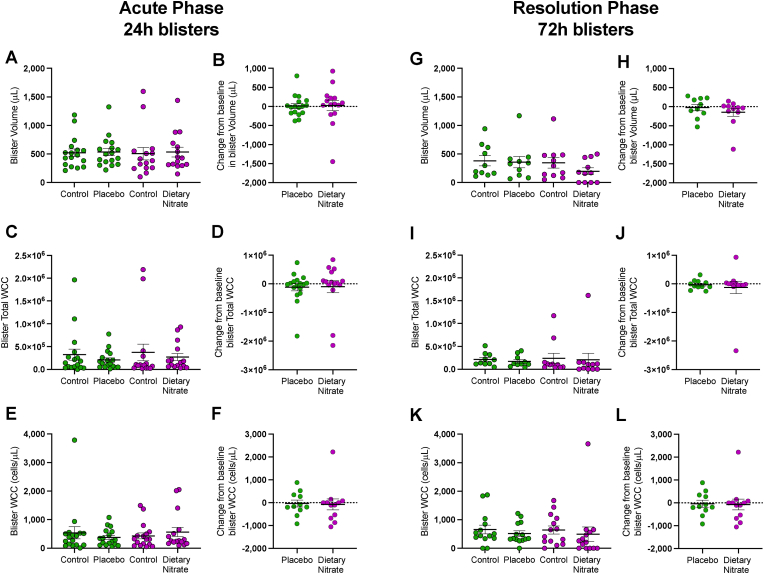
Dietary nitrate treatment does not alter the acute 24h leukocyte response to Cantharidin but does accelerate resolution. Data is shown as scatter with the mean ± SEM indicated at the 24h timepoint for n = 17 volunteers with paired samples in the placebo limb and in the dietary nitrate limb N = 14, and then for the 72h blisters with n = 11 paired samples for the Placebo and N = 12 for the dietary nitrate. No significant differences found in any parameters using paired *t*-test for within group comparison and unpaired *t*-test for comparison of change from baseline.

***Inorganic nitrate accelerates resolution of localised skin blisters*.** Following application of cantharidin, blisters were evident at 24-h (acute phase) and 72-h (resolution phase) in all participants. There was no evidence of a change in circulating leukocyte numbers in either placebo or dietary nitrate-treated volunteers (Table 3). A total of 17 volunteers in the placebo arm and 18 volunteers in the dietary nitrate intervention group were recruited. Due to blister rupture this provided 17 and 15 matched 24h samples and at 72h blisters 11 and 12 matched blisters in the placebo and dietary nitrate groups respectively. At the acute phase (24h) timepoint 0/17 and 0/15 blisters resolved in the placebo and the dietary nitrate groups respectively (P > 0.9999, two-tailed Fisher's exact test). In contrast at 72-h whilst no blisters resolved in those treated with placebo (0/11), 5 out of 12 blisters were resolved in those treated with dietary nitrate (P = 0.0425, two-tailed Fisher's exact test). Acute phase blisters had a mean blister fluid volume of 514 ± 60.4 μL (n = 32), a total leukocyte count of 3.4 ± 1.0 x 10^5^ cells and 483 ± 11.6 cells/μL. In comparison, the average of 72-h control blisters (n = 21 volunteers) gave a lower mean exudate volume of 362 ± 63.1 μL, a total leukocyte count of 2.3 ± 0.5 x 10^5^ cells and a denser cellular infiltrate of 652 ± 103 cells/μL (Fig. 4). As per previous observations [52] at both timepoints cell count and blister volume from the control untreated blisters were correlated (Fig. S7). No statistically significant differences were observed within or for between group comparisons at either the 24h or 72h blisters in these measures.

**Fig. 5:**
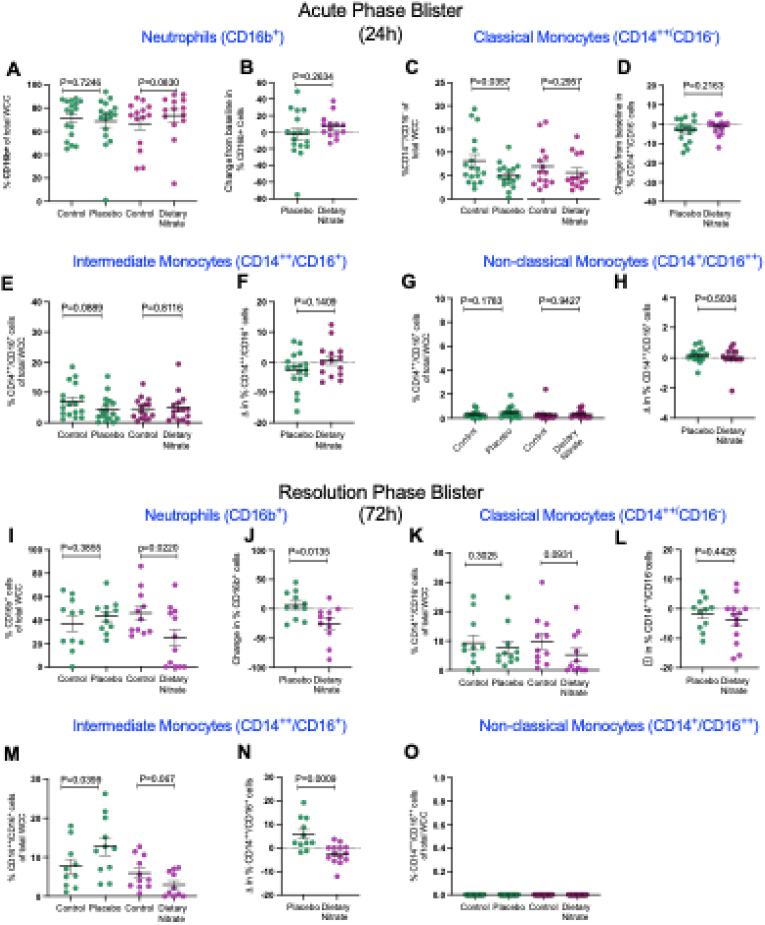
Dietary nitrate treatment leads to significant reductions in the proportion of neutrophils and intermediate pro-inflammatory monocyte levels at 72h. Leukocyte subtypes were identified using specific fluorescence-tagged antibodies and visualised using flow cytometry and are shown as at 24 h A) % neutrophil, B) change in the % neutrophil content from control, C) % classical monocyte and D) change from control, E)% intermediate monocyte and F) change from control, G) % non-classical monocyte and H) change from control. Similar leukocyte sub-populations are shown for the 72h timepoint with neutrophils (I,J) classical monocytes (K,L), intermediate monocytes (M,N) and non-classical monocytes (O). Data is shown as scatter with the mean ± SEM indicated at the 24h timepoint for n = 17 volunteers with paired samples in the placebo limb and in the dietary nitrate limb N = 14, and then for the 72h blisters with n = 11 paired samples for the Placebo and N = 12 for the dietary nitrate. Statistical analysis conducted using paired *t*-test for within group comparison and unpaired *t*-test for comparison of change from baseline.

**Fig. 6:**
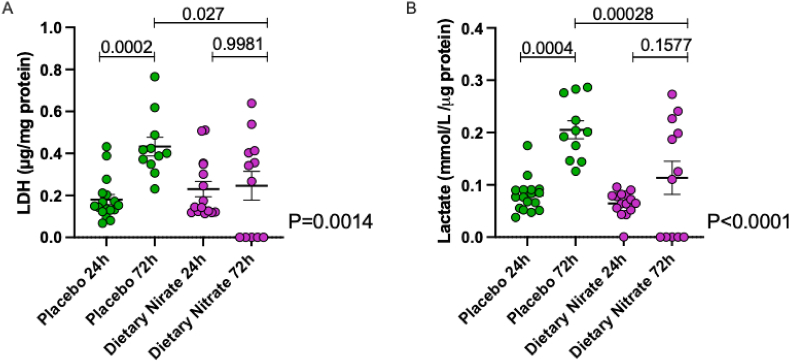
Dietary nitrate treatment is associated with an accelerated resolution of inflammation as reflected by a faster return to a low metabolism setting. Data shows the lactate dehydrogenase activity (LDH, A) and lactate (B) levels ion the blister supernatant and is shown as scatter with the mean ± SEM indicated at the 24h timepoint for n = 17 volunteers in the placebo arm and N = 14 in the dietary nitrate arm, and then for the 72h blisters with n = 11 for the Placebo and N = 12 for dietary nitrate. Statistical significance determined using one-way ANOVA, with Bonferroni's post hoc analysis for multiple group comparison.

**Fig. 7:**
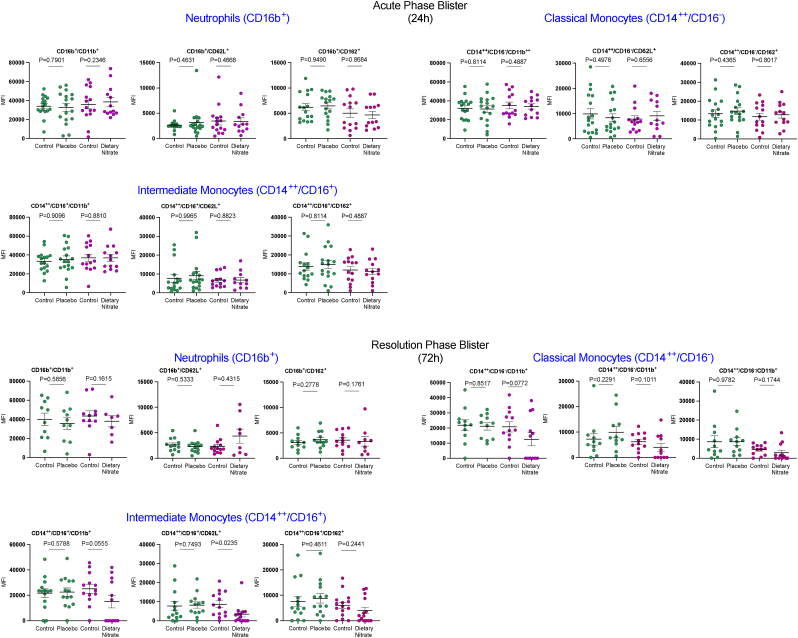
**Dietary nitrate attenuates the activation state of inflammatory and intermediate monocytes during the resolution stage of inflammation whilst having no impact upon neutrophil and monocyte activation state during the acute 24 h response to cantharidin.** Data is shown as scatter with the mean ± SEM indicated at the 24h timepoint for n = 17 volunteers with paired samples in the placebo limb and N = 14 in the dietary nitrate limb. For the 72h blisters data is shown for N = 11 paired samples for the Placebo and N = 12 for the dietary nitrate limb. Data shows the expression of the CD11b, CD62L and CD162 activation markers on leukocyte subtypes identified within blister fluid in volunteers prior to and following treatment with placebo or dietary nitrate treatment. Leukocyte subtypes were identified using specific fluorescence-tagged antibodies and visualised using flow cytometry and expression shown as mean fluorescence intensity (MFI) for each marker. Statistical analysis conducted using paired *t*-test for within group comparison.

***Inorganic nitrate influences localised inflammatory responses***. Acute phase (24h) blisters showed a predominant neutrophil infiltrate (70.0 %), followed by 9.6 % CD14^++^/CD16^-^ classical (inflammatory monocytes) and 5.6 % CD14^+^/CD16^++^ intermediate monocytes. Whilst there were no differences in leukocyte sub-population proportions at the 24h timepoint, at the resolution 72h timepoint there were statistically significantly reduced proportions of neutrophils and intermediate monocytes with dietary nitrate treatment (Fig. 5); a profile supporting the contention of accelerated resolution that likely is precipitated by the improved clearance of dying (apoptotic) cells. Further support for this is provided by the assessment of LDH activity and lactate levels which are measurements of cell death and metabolic activity associated with active apoptosis respectively [53]. In the placebo arm an increase in LDH activity and lactate at 72h compared to 24h occurred, indicating an increase in glycolytic metabolic activity that is expected during an immune response but also confirming that inflammation was underway. However, in the dietary nitrate treated group this rise overall was absent and the levels at 72h statistically significantly lower than the placebo arm (Fig. 6). These observations support the view that resolution had already been initiated and that the inflammation was abating. Further flow cytometry analysis demonstrated that, whilst at the 24h timepoint there was no difference in the activation state of any of the leukocyte sub-populations, there was a substantial and statistically significant generalised suppression in the expression levels of CD11b, CD162 and CD62L in the intermediate and inflammatory monocyte sub-populations at 72h (Fig. 7). A robust inflammatory cytokine/chemokine response was observed in the blisters at 24h (Fig. S8) that was still evident at 72h. There were no statistically significant differences in any mediator assessed between placebo and dietary nitrate treatment except for marginal statistically significant increase in TNFα in placebo treated volunteers within the 24h blister compared to its matched control (Figs. S9 and S10).

**Fig. 8:**
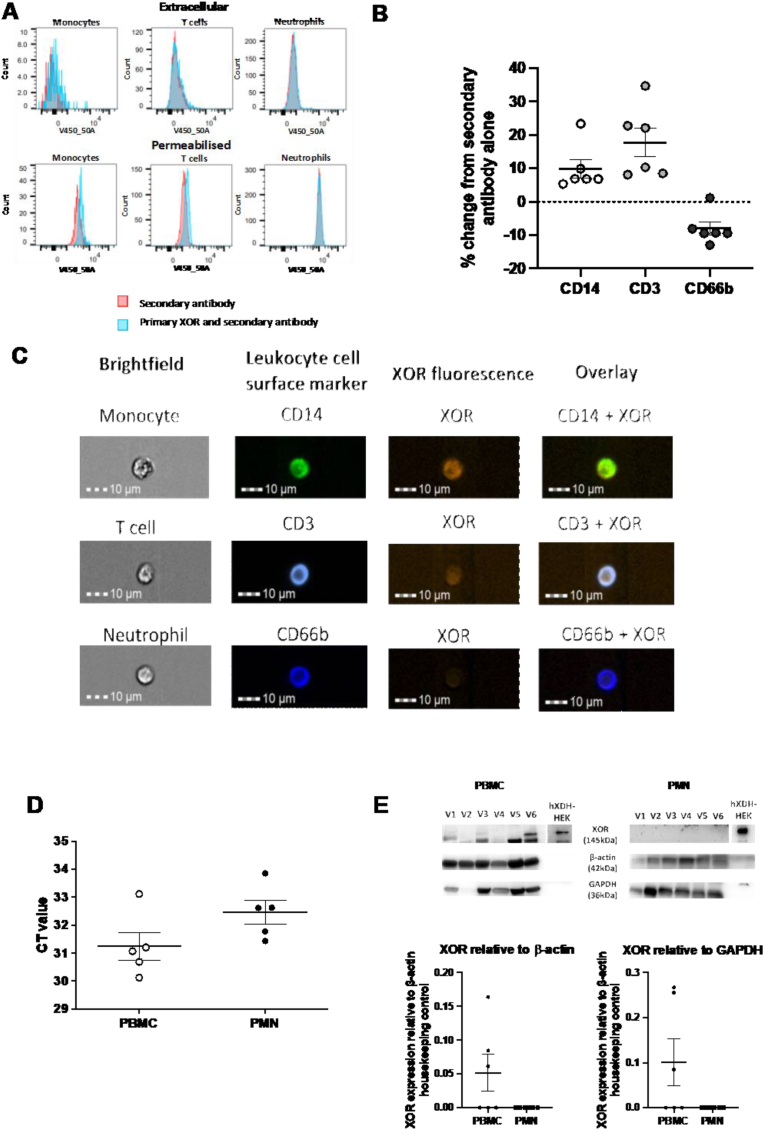
**XOR expression and activity evident in PBMCs.** XOR is absent on the leukocyte cell surface but is present intracellularly in CD14^+^ (monocytes) and CD3^+^ cells (T cells) and absent on CD66b^+^ cells (neutrophils) in healthy individuals. A) Representative histograms of flow cytometry show a rightward shift in permeabilised monocytes and T cells, that is absent in neutrophils and B) shows quantification of this. C) shows images acquired on Image stream confirming the flow cytometry observations. Isolated leukocyte populations were subjected to qPCR (D) and Western blotting (E) PBMCs = monocytes and T cells n = 6. All data is shown of n = 6 healthy volunteers with individual volunteers represented as single points or with the icon V 1–6.

***Leukocyte XOR expression***. It is accepted that the effects of a nitrate intervention are mediated by NO. Previous work by others has suggested that NO exerts effects on most leukocyte subsets. Since in our studies there was a clear leukocyte subset selectivity in the effects of nitrate we reasoned that this may suggest that the effects of nitrite are localised to leukocytes that express a nitrite reductase. There is also substantial evidence implicating xanthine oxidoreductase (XOR) as the primary nitrite reductase in stress settings. Thus, to determine whether this might be the case in acute inflammatory responses we analysed blood leukocytes from healthy volunteers. We established a flow cytometry labelling protocol using a selective antibody for XOR and human embryonic kidney cells stably transfected with the gene for XOR i.e. h*XDH*. Following protocol establishment and validation (Fig. S11) we identified expression of XOR in permeabilised monocytes and T-lymphocytes but not in neutrophils (Fig. 8). This protein expression was matched with qPCR for hXDH demonstrating very low-level expression in PBMC and no expression in isolated neutrophils.

## 4 Discussion

Endothelial dysfunction plays a key role in the pathogenesis of CVD [54], and particularly in hypertensive and atherosclerotic disease. Moreover, the extent of endothelial dysfunction is predictive of future events [55,56] and is triggered by, but also amplifies, the systemic inflammation characteristic of worsening CVD [54]. Despite this association, and despite positive evidence indicating that anti-inflammatory strategies improve CV risk, therapeutics that target inflammation are scarce. This is almost entirely due to the on-target, but ***unwanted***, side effects (increased infection) associated with an anti-inflammatory treatment strategy. Our data herein, suggests that dietary intake of inorganic nitrate influences vascular and inflammatory responses with no attenuation of the acute inflammatory response per se, but rather an acceleration of active resolution. This effect is driven by improvements in the balance favouring anti-inflammatory monocyte activity versus pro-inflammatory leukocyte subsets. In addition, we show that this effect of inorganic nitrate is dependent upon its activation via the non-canonical pathway with conversion to nitrite and then to NO, and importantly that a substantial component of this second step is mediated by XOR within the monocyte itself.

As expected, dietary nitrate elevated concentrations of nitrate and nitrite in plasma, urine and saliva, indicating activation of the non-canonical pathway for NO generation. Importantly, we show in the Typhoid-NITRATE study that low-grade systemic inflammation does not interfere with the enterosalivary circuit of inorganic nitrate as reflected by a very similar rise in circulating nitrite levels following inorganic nitrate consumption in the two studies (∼0.5 μM). Similarly, the rise in cGMP levels following dietary nitrate indicate that the conversion of nitrite to NO is unaffected by systemic inflammation. Interestingly, in volunteers receiving the placebo decreased circulating levels of cGMP occurred following typhoid vaccine compared to baseline suggesting that dietary nitrate intervention restored cGMP levels in the setting of an acute inflammation. These findings give confidence that driving the non-canonical pathway is an effective method for elevating bioavailable NO to trigger downstream signalling resulting in elevations of cGMP, despite systemic inflammation.

Typhoid vaccine induced a temporary endothelial dysfunction at 8h, that had resolved by 32h as per previous observations [37]. This dysfunction was localised at the endothelium since the responses to the NO donor GTN were unaffected. Indeed, the GTN response was identical between the groups at baseline, and at 8h and 32h following typhoid vaccine administration, demonstrating the robustness of this measure, and indicates that any effects of the dietary nitrate did not relate to an effect at the level of the smooth muscle. The absence of any effect of 7-day treatment with dietary nitrate on baseline diameter between visit 1 pre-juice and visit 2 post juice ingestion, suggests that in healthy individuals the dietary dose delivered likely has negligible effect upon healthy vascular function. This matches previous evidence in healthy volunteers using an 8 mmol dose demonstrating no effect of inorganic nitrate on FMD responses [51]. These findings support the general consensus at present that inorganic nitrate intervention improves endothelial function specifically where dysfunction is present, such as following an ischaemic insult [16], or in individuals with disease including peripheral vascular disease [57], hypertension [58], hypercholesterolemia [33] and in post-menopausal women [59] and older (>69 years) men [60]. The endothelial dysfunction was associated with a robust systemic inflammation characterised by a decrease in circulating cGMP levels, an increase in WCC and leukocyte differentials, but without substantial measureable increase in hsCRP levels. Previous studies have not measured CRP levels with the typhoid vaccine [50] and herein we show only a very minor elevation at 8h, that was not statistically different between the treatment groups. Raised hsCRP is a common hallmark of CVD progression [61], and a key element of the acute phase response, and is used to track the systemic inflammation that is linked with disease severity [62]. It is possible that the peak in hsCRP occurred following the 8h collection timepoint. Blood sampling perhaps at 12 and 24h timepoints, as used in the clinical setting to track an acute systemic infection [63], may have been useful. Although, it is noteworthy that such additional blood collections would not have been convenient for the volunteers.

Despite this absence of hsCRP, the increased levels of circulating leukocytes and cytokines indicate a clear systemic low-level inflammation. The rise in WCC was predominantly due to an increase in neutrophil numbers and, albeit to a lesser extent, pro-inflammatory intermediate monocytes. Importantly, this increase was associated with elevations in monocyte activation. This change in circulating leukocyte numbers and activity was associated with increases in the levels of key pro-inflammatory cytokines i.e. IL-6 and CCL2, again confirming the low-level systemic inflammatory status. IL-6 is thought to be the primary stimulator for hepatic CRP generation [62], and thus precedes rises in CRP, supporting the suggestion that measurements at later timepoints may have been useful. Importantly, dietary nitrate treatment did not influence the rise in IL-6 but did attenuate levels of the monocyte activation chemokine, CCL2. Such a profile may bode well for future translation. The lack of effect upon IL-6 suggests that the capability to mount an acute innate immune response remains intact with dietary nitrate treatment. Thus may suggest that the issues surrounding an increase in susceptibility to opportunistic infection that undermines the safety of anti-inflammatory therapies [11,61], may not be an issue with dietary nitrate.

In the Typhoid-NITRATE study, individuals treated with dietary nitrate expressed elevated levels of key anti-inflammatory cytokines, namely TGFβ [64,65] and IL-35 [66, 67], although not IL-10 [68, 69]. These cytokines have been implicated as important immunosuppressors driving monocyte/macrophages to an anti-inflammatory phenotype and hence accelerating recovery and protecting endothelial function. The lack of an effect on IL-10 levels is surprising particularly since our own previous studies in atherosclerotic mouse models identify this cytokine as a key target in the beneficial effects of inorganic nitrate [24]; an observation supported by studies in other rodent models of CVD [70,71]. The reason for this difference is not entirely clear but may relate to the need for capture of distinct timepoints and species differences.

To interrogate more closely the impact of inorganic nitrate on the acute inflammatory response at a site of inflammation we used the cantharidin-blister model. As above inorganic nitrate did not impair the acute response to the inflammatory trigger with similar levels of inflammatory cell numbers and leukocyte sub-populations within the 24h blisters in both treatment arms. However, there was a clear acceleration of recovery in those receiving dietary nitrate versus placebo reflected by a greater proportion of resolved blisters at 72h. This was associated with fewer pro-inflammatory ‘intermediate’ monocytes and a reduction in the activation state of neutrophils within the blister. Intermediate monocytes exhibit predominately pro-inflammatory effects with enhanced release of TNFα and IL-1β [72], key cytokines driving the acute immune response, and express a greater propensity to activate and interact with T-cells [73]. A reduction in their number will also lead to a reduction in neutrophil activation, and therefore a shift away from a pro-inflammatory state. This thesis fits with the general reduced activation state of both the inflammatory and intermediate monocytes within blisters at 72h, supporting the view that resolution was accelerated in those receiving inorganic nitrate. Further analysis of the T-cell profiles to assess whether downstream T cell activity is altered would be of value, although there is recognition from previous studies that at least at the 72h timepoint in this cantharidin model the numbers of T-cells within the formed blisters are limited [74].

The 24h blister fluid shows a typical inflammatory profile, with elevated levels of key mediators (TNFα, IL-1β, IL-6, CXCL1, CXCL5, and CCL2) suggesting a neutrophil-driven immune response [52,74]. This profile, indicative of a neutrophil-driven immune response, was followed at 72h, with a shift toward resolution, with a decrease in pro-inflammatory cytokines and a rise in anti-inflammatory mediators, indicating that the immune response is tapering off. The profile of change in this study differs somewhat from previous reports. The potential reason for this discrepancy could lie in the sex of the volunteers since in this study only males were recruited, while others included both males and females [52]. Research has identified notable sex differences in immune responses, which could explain why the temporal cytokine and chemokine profiles observed in our study diverge from those seen in studies involving both sexes [50].

The levels of the cytokines/chemokines measured within the blister fluid did not differ between treatment groups which differs from the observations following systemic inflammation using the typhoid vaccine. The exact reasons for this are uncertain, although it may indicate that the effects of the intervention on cytokine/chemokine production occur at the level of the circulation rather than after the inflammatory cells have been recruited into the extravascular space. However, for most marker analyses, in blister supernatants, we found considerable variability in the measured levels. In many cases, the levels were below the limits of detection, particularly in resolved blisters with zero blister fluid (in 5 of the 12 paired datasets in the nitrate-treated arm). This likely suggests that the study was underpowered for these measures. Additionally, the blister fluid environment is characterized by high protease activity due to the cellular infiltrate, and it is possible that the apparent lack of detection is related to instability in this activated environment. To address this issue a prospective study powered to detect differences in these mediators, using our preliminary dataset for sample size estimations, may be useful.

Another limitation of the blister *in vivo* assay is the limited number of time points that can be assessed in a single individual. It is possible that collecting blisters at an additional time point (e.g., 48h) between 24 and 72h, as done in a recent study assessing the impact of ageing on inflammation, could provide improved insights on mediators of resolution [52], Since leukocyte numbers and activation state were reduced in those treated with inorganic nitrate, we reasoned that this effect may relate to direct actions of NO on the leukocyte. There are observations suggesting that NO down-regulates the expression of adhesion molecules both directly through nitrosation mechanisms and indirectly by elevating cGMP, which leads to an increase in inflammatory cell apoptosis. These processes, in turn, drive monocyte switching and resolution [75,76]. In addition studies, in mice with chronic granulomatous disease, have shown that NO acts to enhance resolution through activation of the RhoGTPase Rac [77]. Since we show rises in cGMP it is possible that this mechanism underlies the effects seen. However, further studies are needed to confirm the NO-cGMP pathway involvement and could include testing of guanylyl cyclase inhibitors, to prevent cGMP synthesis, or alternatively guanylyl cyclase activators to potentiate the effects of the inorganic nitrate intervention.

It has been shown that elevating nitrite levels within the circulation results, within minutes, in tissue nitrite uptake broadly across all cells and tissues [78]. Once within tissues nitrite remains as a relatively stable storage form of NO that can be released once the conditions, and an appropriate nitrite reductase are present. Since XOR has been strongly implicated as the primary nitrite reductase in pathology we assessed whether leukocytic XOR may be responsible for nitrite reduction in the setting of low-level systemic inflammation. Accordingly, we show cytoplasmic expression of XOR in monocytes (and to a lesser extent lymphocytes) but not neutrophils. We speculate that these findings may resolve the uncertainty surrounding the exact source of anti-inflammatory NO during inflammation resolution [79]. However, we acknowledge that further experiments confirming nitrite reductase activity and sensitivity to XOR inhibition are needed.

As mentioned, within tissues stored nitrite releases NO only once appropriate conditions occur i.e. available nitrite reductase; the activity of which is increased with decreasing pH and/or oxygen tension [31,80]. The acute inflammatory milieu, particularly for instance within cantharidin skin blisters, is a low pH environment, a fact supported in our studies by evidence of elevations of LDH activity and lactate, and has an oxygen tension lower than within the circulation [81,82]. We speculate that the expression of XOR, together with the optimal conditions of low pH and oxygen tension, identifies the marginated monocyte as a potential key site for nitrite-derived NO delivery within an inflammatory site. This localised release of NO likely triggers monocyte switching, which subsequently impacts neutrophil activation and number. Further analysis of the monocyte and T-cell subtypes to determine whether this expression is localised to specific subtypes of each leukocyte type would be of value.

Several limitations of our studies should be acknowledged. The models of local and systemic inflammation are experimental which may differ from those in the clinical setting of chronic CVD. However, many of the pro-inflammatory markers that predict CV event risk in patients were also increased with typhoid vaccination suggesting that extrapolation is justified and that our experimental model is useful, at least in the context of the acute inflammatory response and its consequences. Importantly, in the clinical studies presented here, a key inclusion criterion was male sex. This approach was taken since our own previous work indicated that women express a much faster capacity to resolve acute inflammatory responses than their age-matched counterparts [50]. Thus, the female is not a suitable model for testing the hypothesis that inorganic nitrate might interfere with inflammatory responses by accelerating resolution. However, we have completed an investigation of whether inorganic nitrate might exert positive effects upon vascular function in women receiving vaccination against COVID-19 and we hope to publish those results soon (NCT04889274).

A wide range of diseases, from acute COVID-19 infection to chronic cardiovascular conditions, are characterised by systemic inflammation, reduced NO bioavailability and endothelial dysfunction [83]. In this study, we demonstrate that inorganic nitrate ingestion delivers NO that helps attenuate the vascular dysfunction associated with systemic inflammation. Furthermore, we show that this effect is linked to the upregulation of anti-inflammatory cytokines leading to the polarisation of monocyte/macrophages to adopt an anti-inflammatory phenotype. Thus, in summary, our findings support the use of dietary nitrate as a widely accessible and well tolerated therapeutic option for diseases associated with vascular dysfunction. More importantly, it provides a simple approach to limit the long-term consequences of unresolving inflammation without impairing host defense.

## CRediT authorship contribution statement

**Clement Lau:** Writing – review & editing, Supervision, Investigation, Formal analysis, Data curation. **Christopher P. Primus:** Writing – review & editing, Supervision, Investigation, Formal analysis, Data curation. **Asad Shabbir:** Writing – original draft, Supervision, Investigation, Formal analysis, Data curation. **Ismita Chhetri:** Writing – review & editing, Project administration, Investigation. **Mutsumi Ono:** Writing – review & editing, Project administration, Investigation. **Michael Masucci:** Writing – review & editing, Investigation. **Muhammad Aadil Bin Noorany Aubdool:** Writing – review & editing, Investigation. **Julie Amarin:** Writing – review & editing, Investigation. **Alexander JP. Hamers:** Writing – review & editing, Project administration, Investigation. **Zara Khan:** Writing – review & editing, Investigation. **Nitin Ajit Kumar:** Writing – review & editing, Investigation. **Shanik A. Montalvo Moreira:** Writing – review & editing, Investigation. **Gani Nuredini:** Writing – review & editing, Investigation. **Miski Osman:** Writing – review & editing, Investigation. **Charlotte Whitear:** Writing – review & editing, Investigation. **Tom Godec:** Writing – review & editing, Formal analysis. **Vikas Kapil:** Writing-review and editing, study design. **Gianmichele Massimo:** Writing – review & editing, Investigation. **Rayomand S. Khambata:** Writing – review & editing, Supervision, Methodology, Investigation. **Krishnaraj S. Rathod:** Writing – review & editing, Supervision, Investigation. **Amrita Ahluwalia:** Writing – original draft, Supervision, Resources, Project administration, Funding acquisition, Conceptualization.

## Disclosures

Prof A Ahluwalia is a Director of Heartbeet Ltd and IoNa Therapeutics. A Ahluwalia holds two relevant patents: WO2024160924A1; WO2024160921A1**.** A Ahluwalia has received consultancy fees from Palatin Inc.

## Sources of funding

This work and MO were funded by a 10.13039/501100000274BHF Project Grant PG/19/4/33995.

CL, GM and TG were funded by funded by 10.13039/100015652Barts Charity Cardiovascular Programme (MRG00913)

KSR was funded by a 10.13039/501100000272NIHR Clinical Academic Lecturer (2019).

AH and CP were funded by Derek Willoughby PhD Studentships.

IC was funded by the 10.13039/501100000272NIHR CRN.

## Declaration of competing interest

The authors declare the following financial interests/personal relationships which may be considered as potential competing interests: Prof A Ahluwalia is a Director of Heartbeet Ltd and IoNa Therapeutics. A Ahluwalia holds two relevant patents: WO2024160924A1; WO2024160921A1.

## Data Availability

Data will be made available on request.
