# Supplementary material for: Accelerating inflammatory resolution in humans to improve endothelial function and vascular health: Targeting the non-canonical pathway for NO
Source: Redox Biol. 2025 Mar 28;82:103592. doi: 10.1016/j.redox.2025.103592 (PMC12005330; doi:10.1016/j.redox.2025.103592)
Supplement: Multimedia component 1 [file mmc1.docx]

**Supplementary Methods**

**Full article title**

Accelerating inflammatory resolution in humans to improve endothelial and vascular function: targeting the non-canonical pathway for NO

**Short title**

Lau, Primus, Shabbir *et al.*, Accelerating inflammatory resolution to attenuate endothelial dysfunction

**Co-authors**

Clement Lau^1†^, Christopher P Primus^1†^, Asad Shabbir^1†^, Ismita Chhetri^1^, Mutsumi Ono^1^, Michael Masucci^1^, Muhammad Aadil Bin Noorany Aubdool, Julie Amarin^1^, Alexander JP Hamers^1^, Zara Khan^1^, Nitin Ajit Kumar^1^, Shanik A Montalvo Moreira^1,^ Gani Nuredini^1^, Miski Osman^1^, Charlotte Whitear^1^, Tom Godec^3^, Vikas Kapil^1^, Gianmichele Massimo, Rayomand S Khambata^1^, Krishnaraj S Rathod^1,2^, Amrita Ahluwalia^1,3^

^1^Queen Mary University of London, Barts and The London School of Medicine and Dentistry, London, UK

^2^Department of Cardiology, Barts Heart Centre, 2 St. Bartholomew’s Hospital, Barts Health NHS Trust, London, UK

^3^Barts Cardiovascular Clinical Trials Unit, Queen Mary University of London, Barts and The London School of Medicine and Dentistry, London, UK

^†^C Lau, C Primus and A Shabbir contributed equally to this work

**Corresponding author**

Prof Amrita Ahluwalia BSc PhD

Barts and The London School of Medicine & Dentistry, Queen Mary University of London, London, EC1M 6BQ, UK, ORCID ID; 0000-0001-7626-6399, a.ahluwalia@qmul.ac.uk

***Blood pressure measurement***. Peripheral BP was measured using an Omron 705IT electronic sphygmomanometer. Measurements were performed in the seated position in a temperature controlled and quiet room. BP was measured in triplicate, with the cuff placed on the non-dominant arm in accordance with established guidance^1^.

***PWV and PWA measurements***. PWV and central BP were measured in 62 volunteers (31 in each group) using a Vicorder^®^ device (Skidmore Medical Ltd.) by simultaneously recording a pulse wave from the femoral and carotid arteries using the oscillometric method. Volunteers were rested and remained supine for at least 15 minutes prior to measurement. A cuff was placed around the neck over the carotid artery and the upper thigh. Both cuffs were inflated to 65mmHg, and a pulse time delay was extracted from the oscillometric signal captured by the detector^2^. Aortic length was measured from the sternal notch to the femoral cuff.

***Measurement of brachial artery diameter***. Brachial artery ultrasound was used to measure FMD (62+16 volunteers) and GTN-induced brachial artery dilatation in a sub-cohort of 16 volunteers (8 in each group). In the supine position, the patient’s non-dominant arm was supported on a plinth, and a high-resolution external vascular ultrasound probe (Acuson 128AP/10 with a 7.0-MHz linear array transducer) was used to identify the brachial artery in longitudinal plane. The images were magnified, and the region of interest (ROI) identified using Cardiovascular Suite™ software (Quipu Srl.). The intimal borders were identified, and a pneumatic forearm cuff was inflated to 300mmHg for 5 minutes. Following cuff deflation, the vessel was observed for a further 5 minutes. Brachial artery dilatation was expressed as a percentage change (FMD, %)^3, 4^. With the volunteer in the same position, after return to baseline vessel diameter, 400μg sublingual GTN was administered, and the vessel was observed for 5 minutes. Peak brachial artery diameter was measured and expressed as a percentage increase from baseline (GTN-induced brachial artery dilatation, %).

***Blood sampling***. Whole blood was obtained from a vein in the antecubital fossa using a 21-gague butterfly needle and collected directly into BD Vacutainer^®^ EDTA, SST and citrate containing blood tubes. Haematological analysis, leukocyte differential count and clinical biochemistry, including urea, creatinine, sodium, potassium and urate, were conducted at St. Bartholomew’s Hospital Haematology and Biochemistry department. An additional 4mL of blood was collected in an EDTA tube for blood plasma nitrite and nitrate (NO_x_) measurement. For measurement of NO_x_, samples were centrifuged immediately after blood collection at 1300*g* at 4°C for 10 minutes, after mixing with isobutylmethylxanthine (IBMX, 100μM). 200μL of spun plasma was then filtered using a Microcon^®^ Ultracel YM/3 (3kDa) Filter (Millipore Corporation, Billerica, USA) for 60 minutes at 1300*g* and 4°C for deproteinated NO_x_ quantification. Plasma was pipetted into 1.5mL cryotubes and frozen in liquid nitrogen, before being stored at -80°C for future assessment.

***Urine and saliva sampling***. Clean catch mid-stream urine samples were collected in sterile gallipots, aliquoted into 1.5mL microcentrifuge tubes for NO_x_ quantification. Participants were instructed to allow saliva to drip from their tongue/lower lip into a sterile gallipot. This saliva was aliquoted and spun at 1300*g* for 10 minutes at 4°C, and supernatant stored as above.

***Inflammatory cell flow cytometry***. Flow cytometry was used to assess systemic inflammatory response and blister fluid infiltrate. For circulating cell types 50μl of citrated whole blood was incubated with monoclonal antibodies conjugated to fluorochromes to characterise monocytes, neutrophils and additionally T-cell subtypes (although these data are not shown in this manuscript). The antibodies used were CD14, BD Biosciences Cat# 555397, RRID:AB_395798 ; CD16, BD Biosciences Cat# 555407, RRID:AB_395807; CD16b, BD Biosciences Cat# 550868, RRID:AB_393936. Cellular activation state was determined by the quantification of CD162 (Thermo Fisher Scientific Cat# 17-1629-42, RRID:AB_2043811), CD62L (Thermo Fisher Scientific Cat# 47-0629-42, RRID:AB_1582224) and CD11b (BD Biosciences Cat# 558123, RRID:AB_397043) expression, with the use of their respective isotype controls (CD162, Thermo Fisher Scientific Cat# 17-4724-41, RRID:AB_10598641; CD62L, Thermo Fisher Scientific Cat# 47-4714-80, RRID:AB_1271993; CD11b, BD Biosciences Cat# 558120, RRID:AB_397040). Samples were incubated for 30 minutes and then washed using phosphate buffered saline (PBS) by centrifugation for 5 minutes at 400*g* at 21°C. The supernatant was decanted, and red cell lysis conducted using whole blood lysing kit (Beckman Coulter Ltd.). The tubes were vortexed, and the fixative agent (supplied within the kit) added. Samples were subsequently washed twice more using PBS followed by centrifugation for 5 minutes at 400*g* at 21°C. PBS was added and samples stored at 4^o^C until acquisition using a BD LSRFortessa™ Flow Cytometer, and data recorded using BD FACSDiva™ analysis software.

Blister exudate pellet was mixed with PBS, and the resuspended cells (20μL) incubated with the antibodies above for 30-minutes. Following a single wash, samples were analysed as above.

***Leukocyte XOR expression***. To detect leukocyte XOR expression, blood was collected as above and mixed 1:1 v/v with PBS. Peripheral blood mononuclear cells (PBMCs) and polymorphonuclear cells (PMNs) were isolated using density centrifugation. Histopaque 1077 (Sigma, UK) was layered on top of histopaque 1119 (Sigma, UK) and the diluted blood layered above, followed by centrifugation at 700*g*, 30min, room temp with brake off. PBMCs and PMNs were collected and seeded in a 96 well plate for antibody staining. Cell pellets were incubated in 2% human and donkey serum blocking buffer for 15min and subsequently labelled for anti-human CD14 FITC (BD Biosciences Cat# 555397, RRID:AB_395798), anti-human CD3 APC (BioLegend Cat# 300312, RRID:AB_314048), CD66b (BioLegend Cat# 305114, RRID:AB_2566038) and anti-human xanthine oxidase (1:1000; Abcam Cat# ab133268, RRID:AB_11154903), for 30 minutes at 4^o^C. Cells were washed and the secondary antibody for XO (donkey anti-rabbit IgG preadsorbed, Abcam Cat# ab175649, RRID:AB_2715515) incubated for 30 minutes 4^o^C. Cells were then washed twice, fixed (Intracellular fixation and permeabilisation buffer, Thermofisher, UK) and subsequently stored in PBS before acquisition to assess extracellular expression. In order to assess intracellular expression, cells were permeabilised (Intracellular fixation and permeabilisation buffer, Thermofisher, UK) and the cells incubated in blocking buffer followed by primary XO antibody and secondary antibody as described above. Samples were analysed using a BD LSR Fortessa™ Flow Cytometer, and data recorded using BD FACSDiva™ analysis software. For representative images of leukocytes samples were prepared as above and acquired using Amnis^®^ Imagestream X MK2 and analysed using Ideas software v6.2. Representative histogram images were produced using FlowJo v10.8.1.

***Immunoblotting.*** Human PBMC homogenates were subjected to SDS/PAGE (0.1% w/v) immunoblotting analysis using an anti-human XOR rabbit antibody (1:2000, Abcam 133268 RRID:AB_11154903). Briefly, human PBMC pellets were homogenised in 200µl of buffer consisting of PBS, 1% Triton X, with 5.7μM benzamidine, 1.5μM antipain, 0.15μM aprotinin, 4.2μM leupeptin, 1.5μM pepstatin A and 400μM AEBSF protease inhibitor and mechanically disrupted using a 25-gague needle (0.5mm). The resulting homogenate was centrifuged at 4**°**C for 10 minutes at 14,000 RPM and the supernatant collected. Protein concentration was determined using a Pierce**^®^** BCA Protein Assay Kit (Thermoscientific, UK) according to manufacturer guidelines. 200μg of protein for each sample was prepared for electrophoresis and loaded onto an 8-16% Mini-Protean TGX gels (Bio-rad). Following this, electrophoresed proteins were transferred via a semi-dry transfer method to a 0.2µm nitrocellulose membrane (Amersham™ Proton™). Red Ponceau solution was used to confirm transfer prior to overnight incubation with primary antibody. The membrane was then washed and incubated with an anti-rabbit secondary antibody (1:5000, Invitrogen RRID: AB_2536381) for 1 hour. The nitrocellulose membrane was exposed to a 1:1 v/v solution of Clarity Western ECL Substrate (Bio-rad) for 5 minutes and chemiluminescence quantified and analysed using FluorChem E software (ProteinSimple). Two housekeeping proteins, GAPDH (Thermo Fisher Scientific Cat# AM4300 (RRID:AB_2536381)) and β-actin (Millipore Cat# MAB1501 (RRID: AB_2223041)), were also measured for internal normalization.

***qPCR assessment of leukocyte XOR expression***. RNA from PBMCs and PMNs was extracted, using Nucleospin RNA extraction kit (Macherey Nagel, Germany), according to manufacturer guidelines. cDNA was synthesised and qPCR analysis carried out with SYBR green (ThermoFisher Scientific, UK), using specifically designed primers for hXDH 5’-CTCAGTCAGCCTCTCGCCAT-3’ and 5’-TATCCACGTCACACGCTCCC-3’. qRT-PCR was performed using an ABI Quantstudio 7 sequence detection system.

***Inflammatory mediator analysis****.* Cytokine and chemokine expression profiles, in acute and resolution phase blisters following cantharidin application and in plasma collected from the volunteers before and 8h following Typhoid vaccine administration, were analysed blind to treatment allocation by an external commercial organisation (LaboSpace) using a bead array (IDEXX ProCyte Dx Haematology Analyser) measuring: IL-1β, TNFα, IFNγ, IL-1α, IL-6, CXCL5/NEA78, IL-8/CXCL8, MCP-1/CLL2, IL-21, IL 17A, IL-17E/IL-25, IL17F, IL-23, MIP-2/CXCL2, SDF-1/CXCL12, CX_3_CL_1_, CXCL1, MIP-1α/CCL3, TGFβ, IL-10 and IL-35.

Soluble CD62L was quantified using a sandwich enzyme linked immunosorbent assay (ELISA) (human L-selectin/CD62L, R&D Systems, catalogue DY728) and hsCRP using a sandwich ELISA (Human CRP ELISA Kit, catalogue KHA0031) as per the manufacturer’s instructions.

As an indirect measure of apoptosis in blisters we assessed the levels of lactate dehydrogenase (LDH) activity (CyQuant LDH cytoxicity assay kit, C20300, Invitrogen, UK) in the cell free blister fluid supernatant as per the manufacturer’s instruction. Using the supplied LDH positive control we constructed a standard curve and then used this curve to assess LDH activity of the diluted blister fluid. Supernatants were diluted 1 in 10 or 1 in 100 and LDH activity expressed relative to protein concentration. To assess lactate levels within the blister fluid the L-lactate assay kit (ab65331, Abcam, UK) was used. 1.5μL of sample was loaded and the volume adjusted to 50μL as per manufacturer instructions. Samples that were above the highest standard were diluted 1 in 10 and rerun. Samples did not require a deproteinization step.

***Assessment of nitrite, nitrate and cGMP levels and leukocyte nitrite reductase activity***. Ozone chemiluminescence was conducted for quantification of plasma, urine and saliva nitrite (NO_2_^-^) and NO_x_ as previously described^5, 6^. Nitrate (NO_3_^-^) was determined by the subtraction of NO_2_^-^ from NO_x_. Nitrite-reductase activity was measured as previously described^7^. PBMC pellets were homogenised with homogenization buffer devoid of Triton X-100. Experiments were performed in a sealed 10mL glass reaction chamber containing citric acid/Na₂HPO₄ buffer at pH 6.8 (representing acidosis), and NaNO_2_ (10‐1000 μM) in a total volume of 1 ml. This solution was bubbled with nitrogen gas (100%) via an NO scrubbing air filter (Sievers, USA). Headspace NO concentration was measured in parts per billion by continuous sampling using a 280A Nitric Oxide Analyzer (Sievers, USA). The impact of biological tissue on NO production from NO_2_^-^ , above that achieved in the absence of protein, was determined by the addition of 200μg of protein and measurement of chemiluminescent signal of NO over 2 minutes, calculating the rate of NO production (nmol g protein^-1^ s^-1^) from the area under the curve.

cGMP concentration in plasma samples were quantified using an Enzymeimmunoassay Biotrak kit (GE Healthcare, catalogue RPN226) as per manufacturer’s instructions.

1. Williams B, Poulter NR, Brown MJ, Davis M, McInnes GT, Potter JF*, et al.* British Hypertension Society guidelines for hypertension management 2004 (BHS-IV): summary. BMJ 2004;**328**(7440):634-40.

2. Hickson SS, Butlin M, Broad J, Avolio AP, Wilkinson IB, McEniery CM. Validity and repeatability of the Vicorder apparatus: a comparison with the SphygmoCor device. Hypertens Res 2009;**32**:1079-1085.

3. Celermajer DS, Sorensen KE, Gooch VM, Spiegelhalter DJ, Miller OI, Sullivan ID*, et al.* Non-invasive detection of endothelial dysfunction in children and adults at risk of atherosclerosis. Lancet 1992;**340**(8828):1111-1115.

4. Thijssen DHJ, Bruno RM, van Mil A, Holder SM, Faita F, Greyling A*, et al.* Expert consensus and evidence-based recommendations for the assessment of flow-mediated dilation in humans. Eur Heart J 2019;**40**(30):2534-2547.

5. Velmurugan S, Gan JM, Rathod KS, Khambata RS, Ghosh SM, Hartley A*, et al.* Dietary nitrate improves vascular function in patients with hypercholesterolemia: a randomized, double-blind, placebo-controlled study. Am J Clin Nutr 2016;**103**(1):25-38.

6. Buga GM, Griscavage JM, Rogers NE, Ignarro LJ. Negative feedback regulation of endothelial cell function by nitric oxide. Circ.Res. 1993;**73**:808-812.

7. Ghosh SM, Kapil V, Fuentes-Calvo I, Bubb KJ, Pearl V, Milsom AB*, et al.* Enhanced Vasodilator Activity of Nitrite in Hypertension: Critical Role for Erythrocytic Xanthine Oxidoreductase and Translational Potential. Hypertension 2013;**61**(5):1091-1102.
