# Supplementary material for: Accelerating inflammatory resolution in humans to improve endothelial function and vascular health: Targeting the non-canonical pathway for NO
Source: Redox Biol. 2025 Mar 28;82:103592. doi: 10.1016/j.redox.2025.103592 (PMC12005330; doi:10.1016/j.redox.2025.103592)
Supplement: Multimedia component 2 [file mmc2.pptx]

## Slide 1
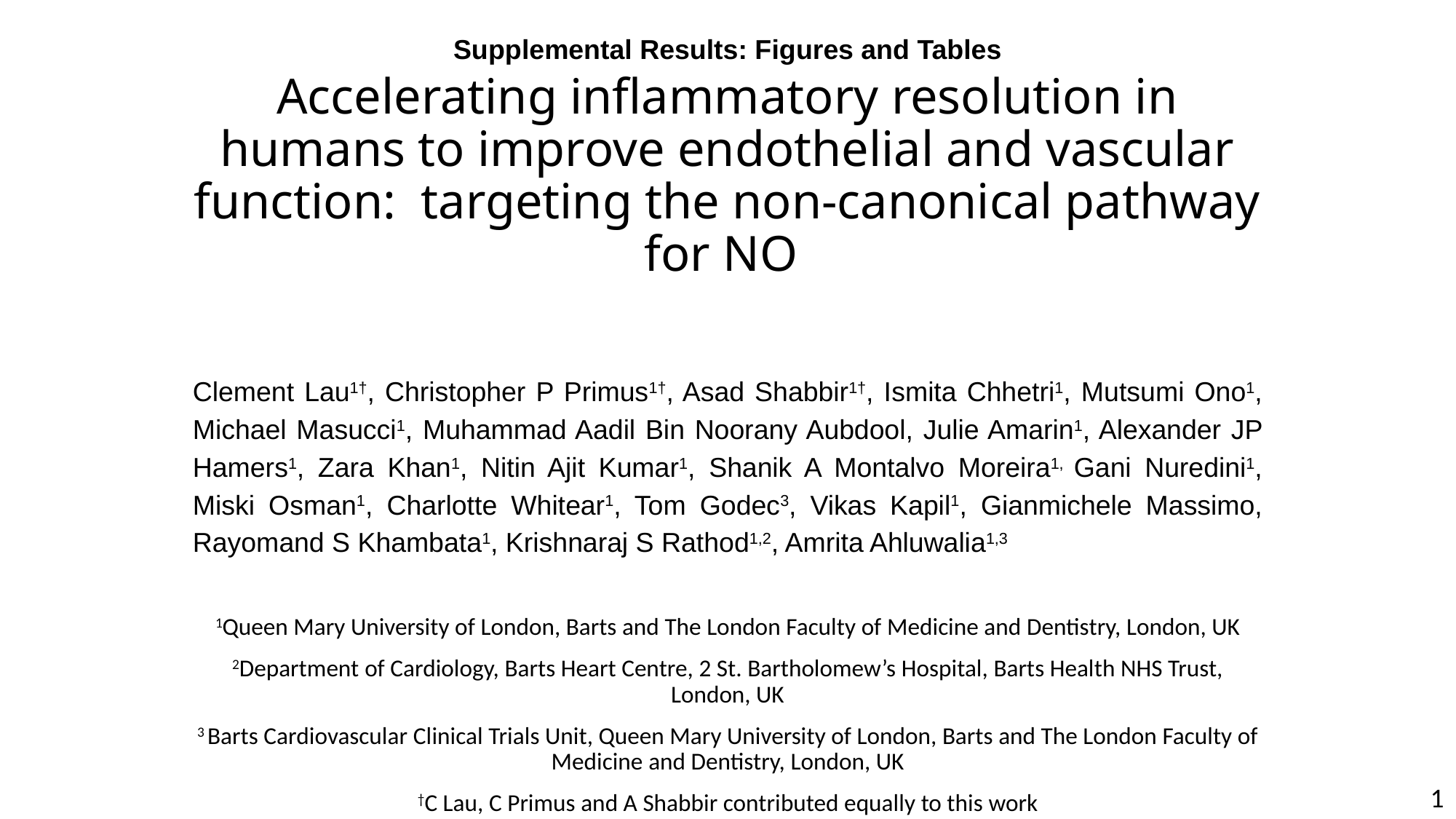

# Supplemental Results: Figures and Tables Accelerating inflammatory resolution in humans to improve endothelial and vascular function: targeting the non-canonical pathway for NO
Clement Lau1†, Christopher P Primus1†, Asad Shabbir1†, Ismita Chhetri1, Mutsumi Ono1, Michael Masucci1, Muhammad Aadil Bin Noorany Aubdool, Julie Amarin1, Alexander JP Hamers1, Zara Khan1, Nitin Ajit Kumar1, Shanik A Montalvo Moreira1, Gani Nuredini1, Miski Osman1, Charlotte Whitear1, Tom Godec3, Vikas Kapil1, Gianmichele Massimo, Rayomand S Khambata1, Krishnaraj S Rathod1,2, Amrita Ahluwalia1,3
1Queen Mary University of London, Barts and The London Faculty of Medicine and Dentistry, London, UK
2Department of Cardiology, Barts Heart Centre, 2 St. Bartholomew’s Hospital, Barts Health NHS Trust, London, UK
3 Barts Cardiovascular Clinical Trials Unit, Queen Mary University of London, Barts and The London Faculty of Medicine and Dentistry, London, UK
†C Lau, C Primus and A Shabbir contributed equally to this work
1

## Slide 2
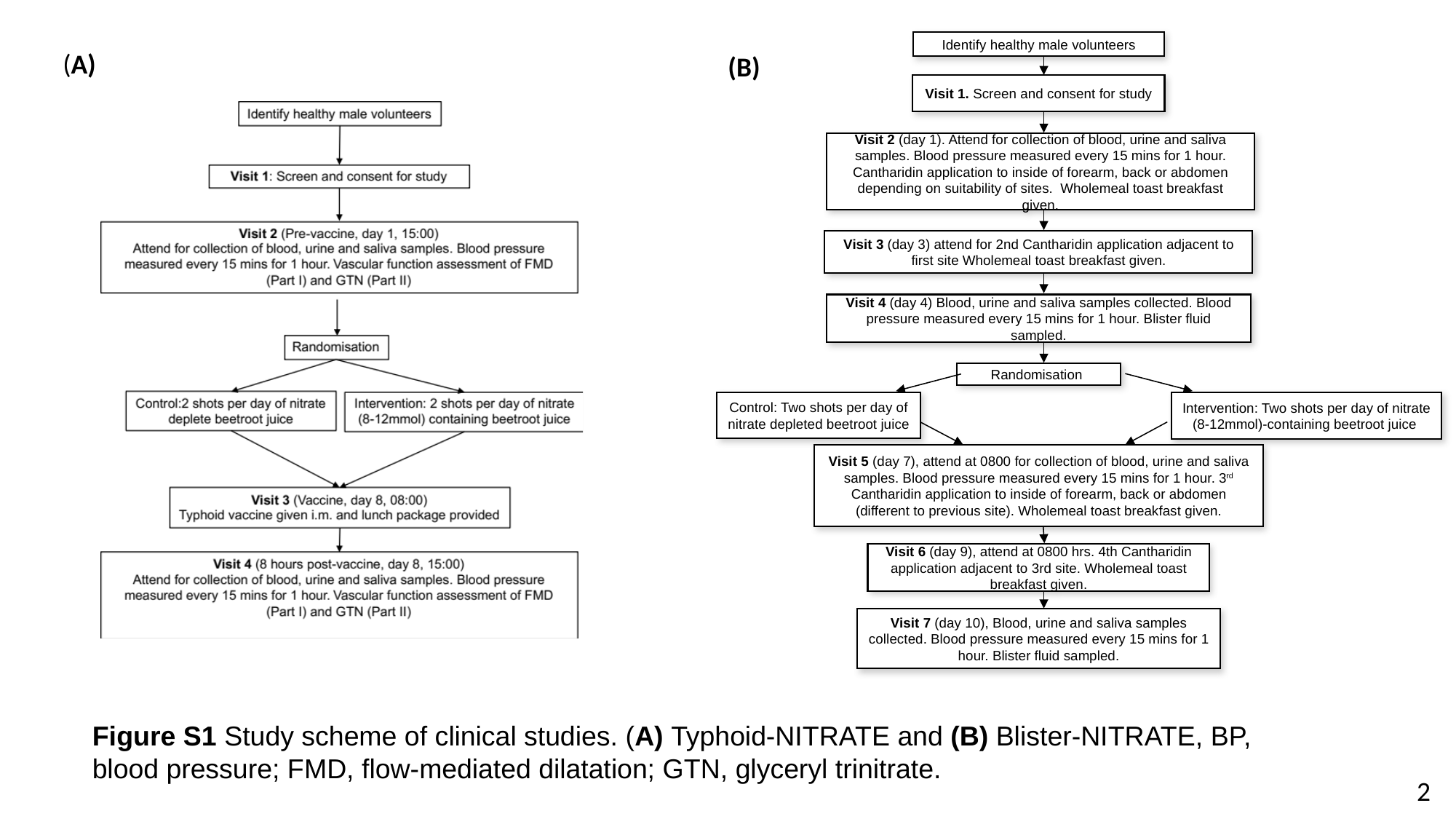

Identify healthy male volunteers
Visit 1. Screen and consent for study
Visit 2 (day 1). Attend for collection of blood, urine and saliva samples. Blood pressure measured every 15 mins for 1 hour. Cantharidin application to inside of forearm, back or abdomen depending on suitability of sites. Wholemeal toast breakfast given.
Visit 3 (day 3) attend for 2nd Cantharidin application adjacent to first site Wholemeal toast breakfast given.
Visit 4 (day 4) Blood, urine and saliva samples collected. Blood pressure measured every 15 mins for 1 hour. Blister fluid sampled.
Randomisation
Intervention: Two shots per day of nitrate (8-12mmol)-containing beetroot juice
Control: Two shots per day of nitrate depleted beetroot juice
Visit 5 (day 7), attend at 0800 for collection of blood, urine and saliva samples. Blood pressure measured every 15 mins for 1 hour. 3rd Cantharidin application to inside of forearm, back or abdomen (different to previous site). Wholemeal toast breakfast given.
Visit 6 (day 9), attend at 0800 hrs. 4th Cantharidin application adjacent to 3rd site. Wholemeal toast breakfast given.
Visit 7 (day 10), Blood, urine and saliva samples collected. Blood pressure measured every 15 mins for 1 hour. Blister fluid sampled.
(A)
(B)
Figure S1 Study scheme of clinical studies. (A) Typhoid-NITRATE and (B) Blister-NITRATE, BP, blood pressure; FMD, flow-mediated dilatation; GTN, glyceryl trinitrate.
2

## Slide 3
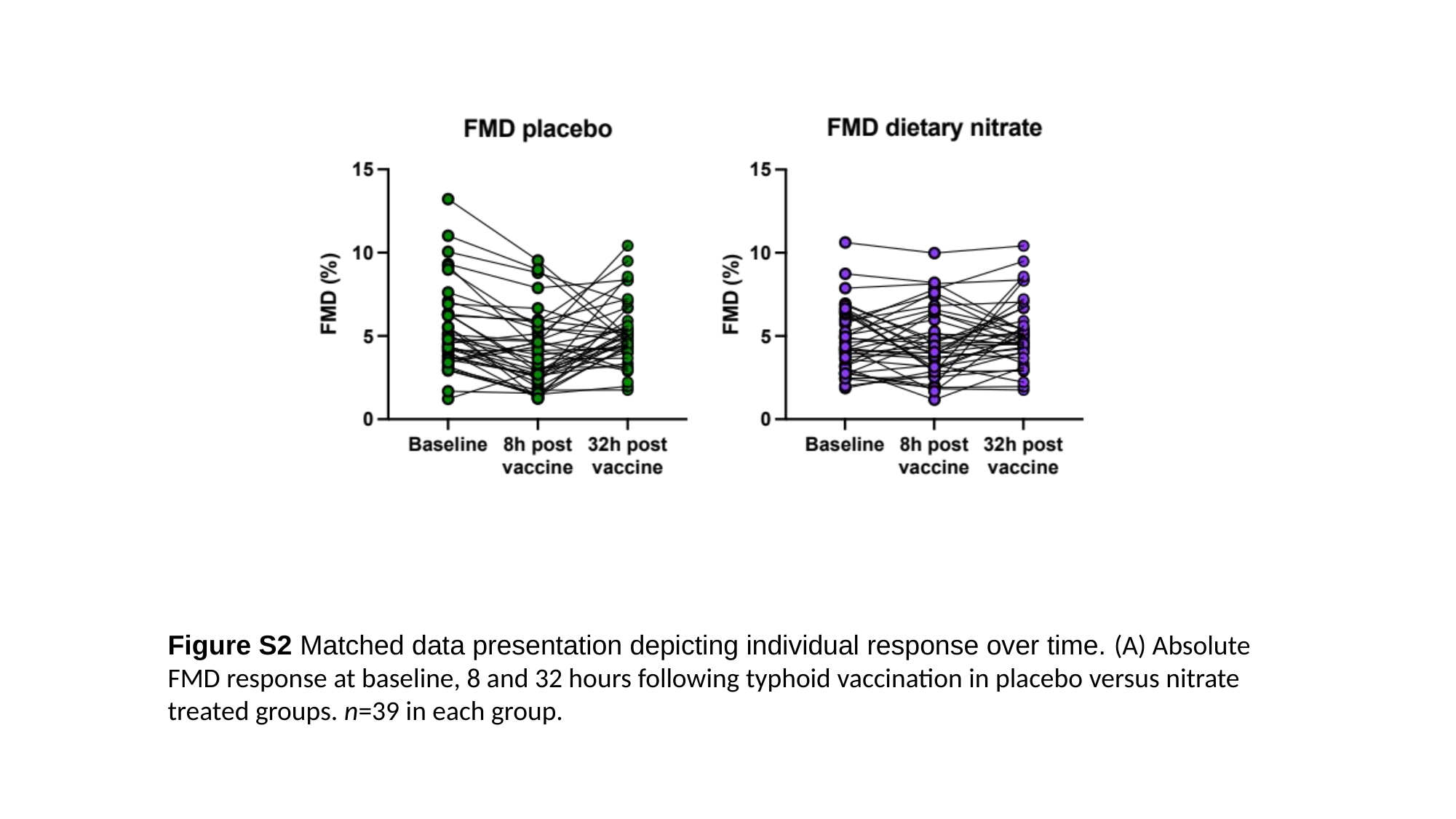

Figure S2 Matched data presentation depicting individual response over time. (A) Absolute FMD response at baseline, 8 and 32 hours following typhoid vaccination in placebo versus nitrate treated groups. n=39 in each group.

## Slide 4
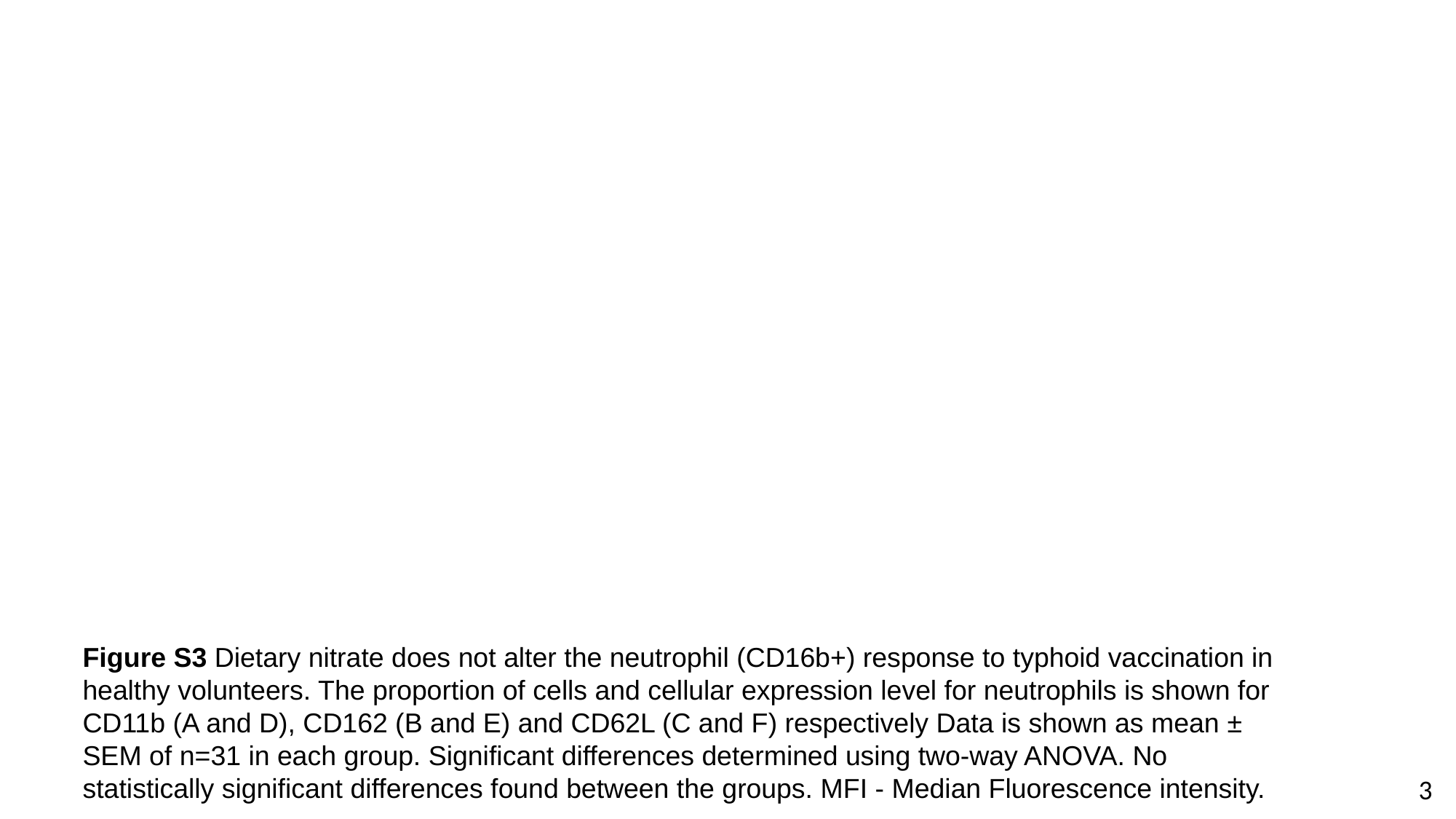

Figure S3 Dietary nitrate does not alter the neutrophil (CD16b+) response to typhoid vaccination in healthy volunteers. The proportion of cells and cellular expression level for neutrophils is shown for CD11b (A and D), CD162 (B and E) and CD62L (C and F) respectively Data is shown as mean ± SEM of n=31 in each group. Significant differences determined using two-way ANOVA. No statistically significant differences found between the groups. MFI - Median Fluorescence intensity.
3

## Slide 5
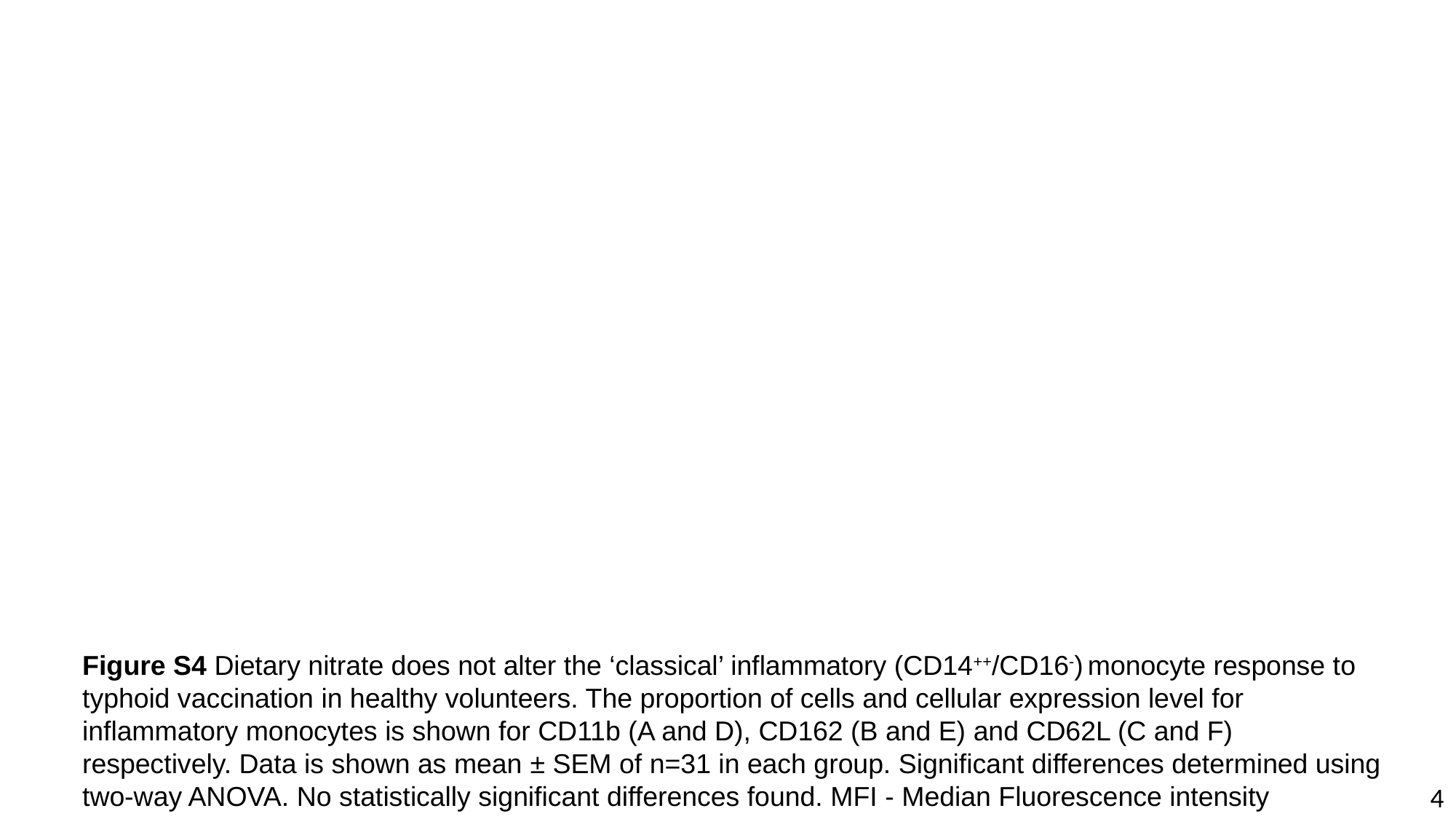

Figure S4 Dietary nitrate does not alter the ‘classical’ inflammatory (CD14++/CD16-) monocyte response to typhoid vaccination in healthy volunteers. The proportion of cells and cellular expression level for inflammatory monocytes is shown for CD11b (A and D), CD162 (B and E) and CD62L (C and F) respectively. Data is shown as mean ± SEM of n=31 in each group. Significant differences determined using two-way ANOVA. No statistically significant differences found. MFI - Median Fluorescence intensity
4

## Slide 6
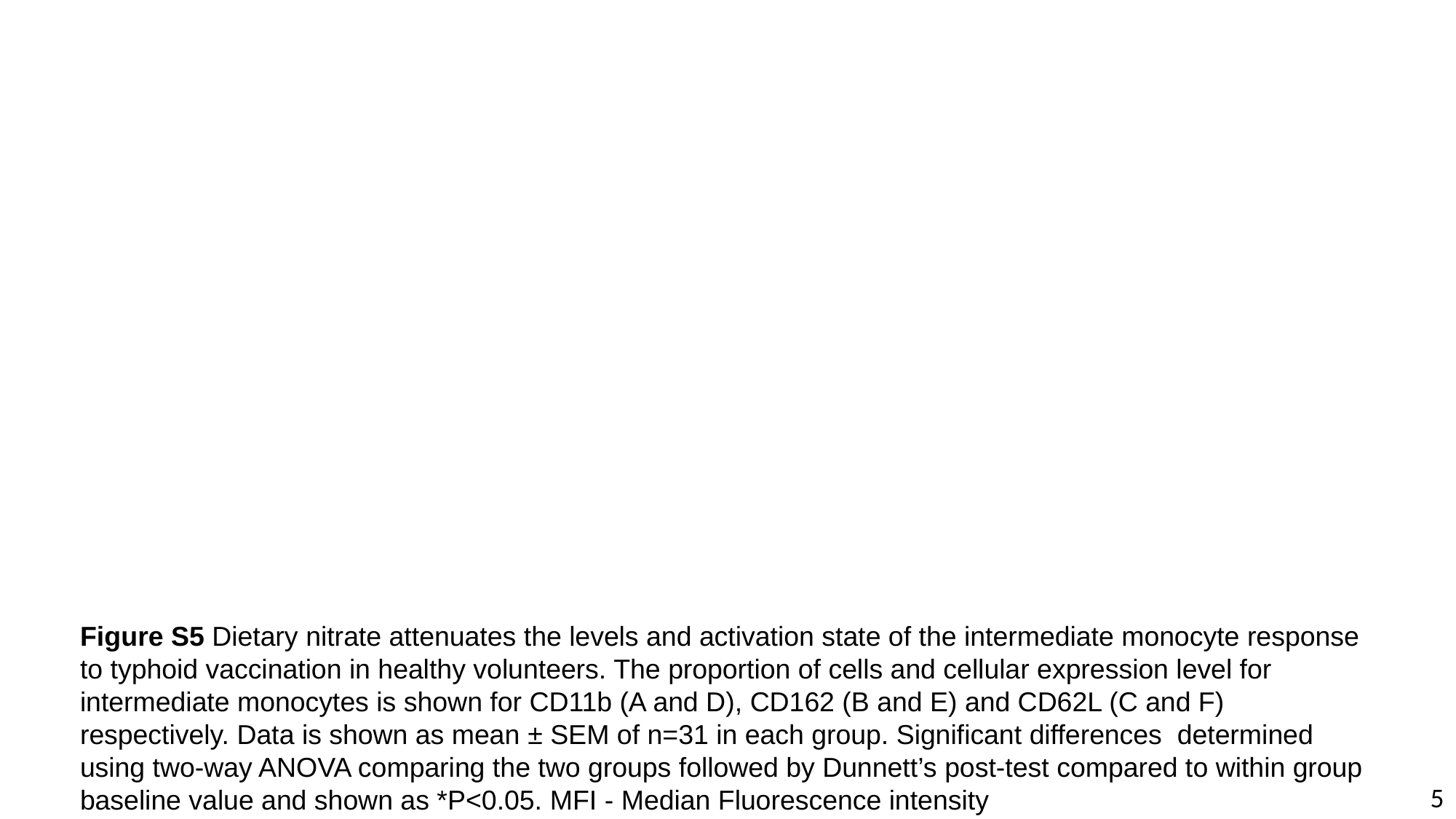

Figure S5 Dietary nitrate attenuates the levels and activation state of the intermediate monocyte response to typhoid vaccination in healthy volunteers. The proportion of cells and cellular expression level for intermediate monocytes is shown for CD11b (A and D), CD162 (B and E) and CD62L (C and F) respectively. Data is shown as mean ± SEM of n=31 in each group. Significant differences determined using two-way ANOVA comparing the two groups followed by Dunnett’s post-test compared to within group baseline value and shown as *P<0.05. MFI - Median Fluorescence intensity
5

## Slide 7
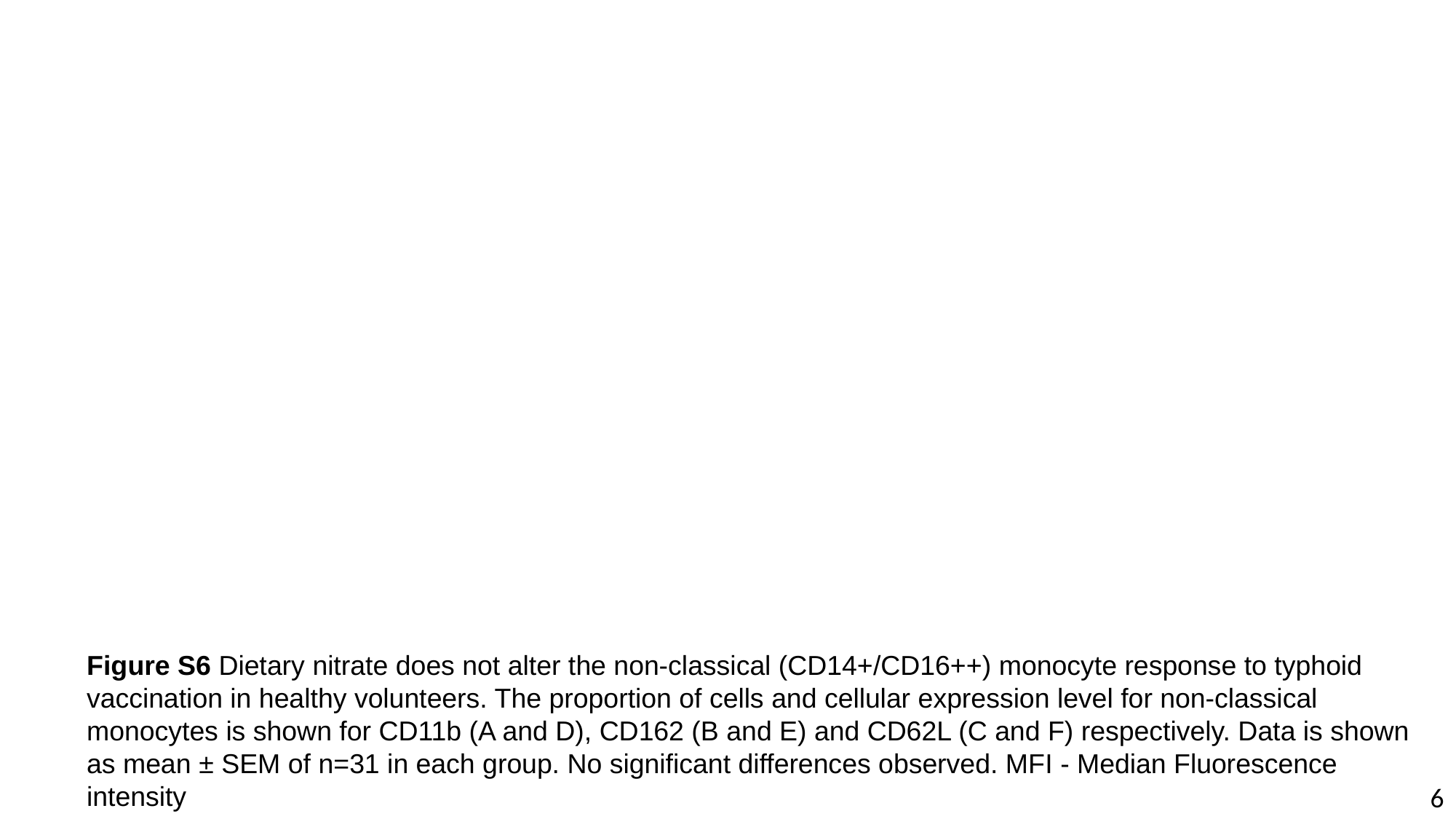

Figure S6 Dietary nitrate does not alter the non-classical (CD14+/CD16++) monocyte response to typhoid vaccination in healthy volunteers. The proportion of cells and cellular expression level for non-classical monocytes is shown for CD11b (A and D), CD162 (B and E) and CD62L (C and F) respectively. Data is shown as mean ± SEM of n=31 in each group. No significant differences observed. MFI - Median Fluorescence intensity
6

## Slide 8
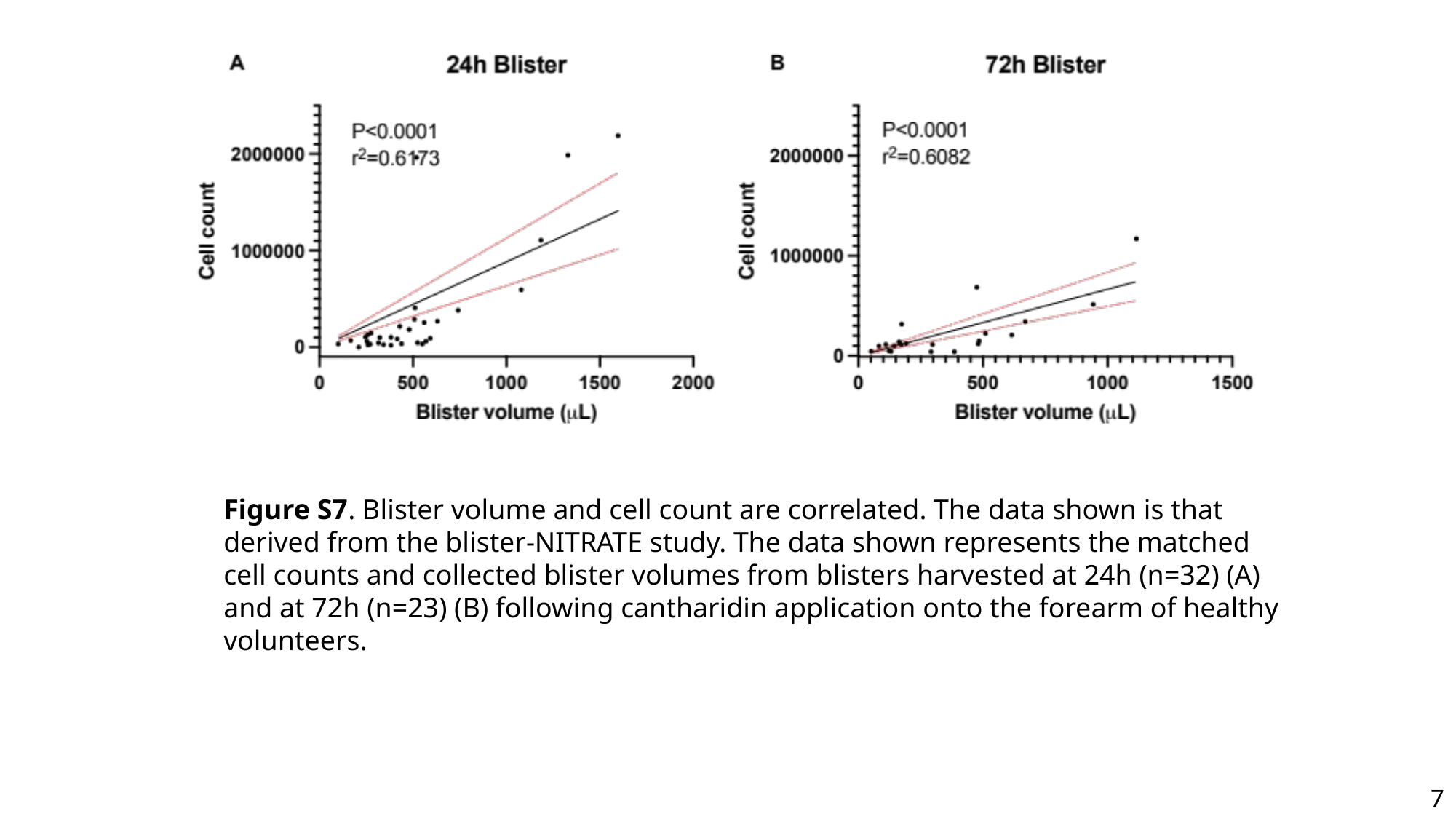

Figure S7. Blister volume and cell count are correlated. The data shown is that derived from the blister-NITRATE study. The data shown represents the matched cell counts and collected blister volumes from blisters harvested at 24h (n=32) (A) and at 72h (n=23) (B) following cantharidin application onto the forearm of healthy volunteers.
7

## Slide 9
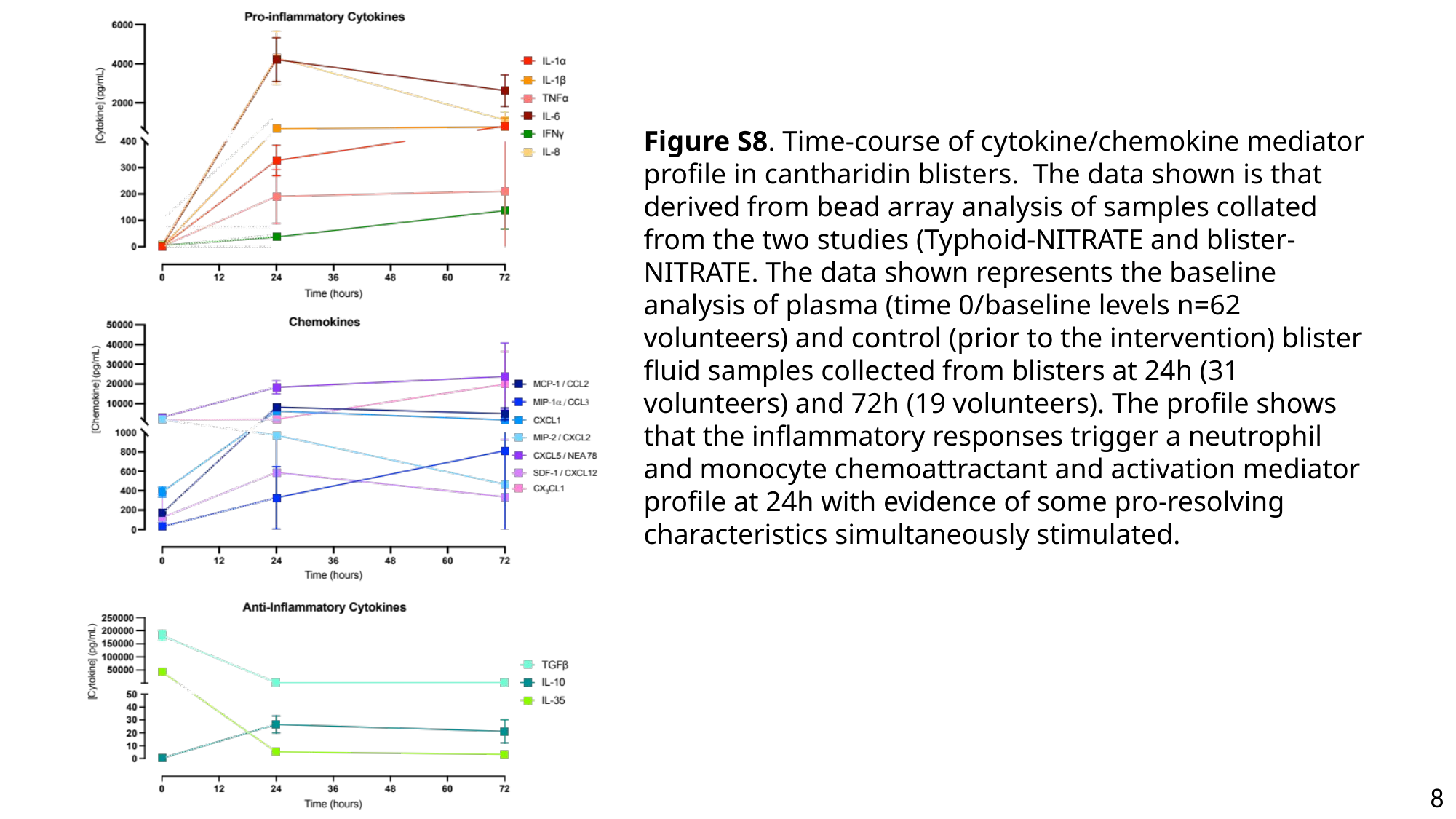

Figure S8. Time-course of cytokine/chemokine mediator profile in cantharidin blisters. The data shown is that derived from bead array analysis of samples collated from the two studies (Typhoid-NITRATE and blister-NITRATE. The data shown represents the baseline analysis of plasma (time 0/baseline levels n=62 volunteers) and control (prior to the intervention) blister fluid samples collected from blisters at 24h (31 volunteers) and 72h (19 volunteers). The profile shows that the inflammatory responses trigger a neutrophil and monocyte chemoattractant and activation mediator profile at 24h with evidence of some pro-resolving characteristics simultaneously stimulated.
8

## Slide 10
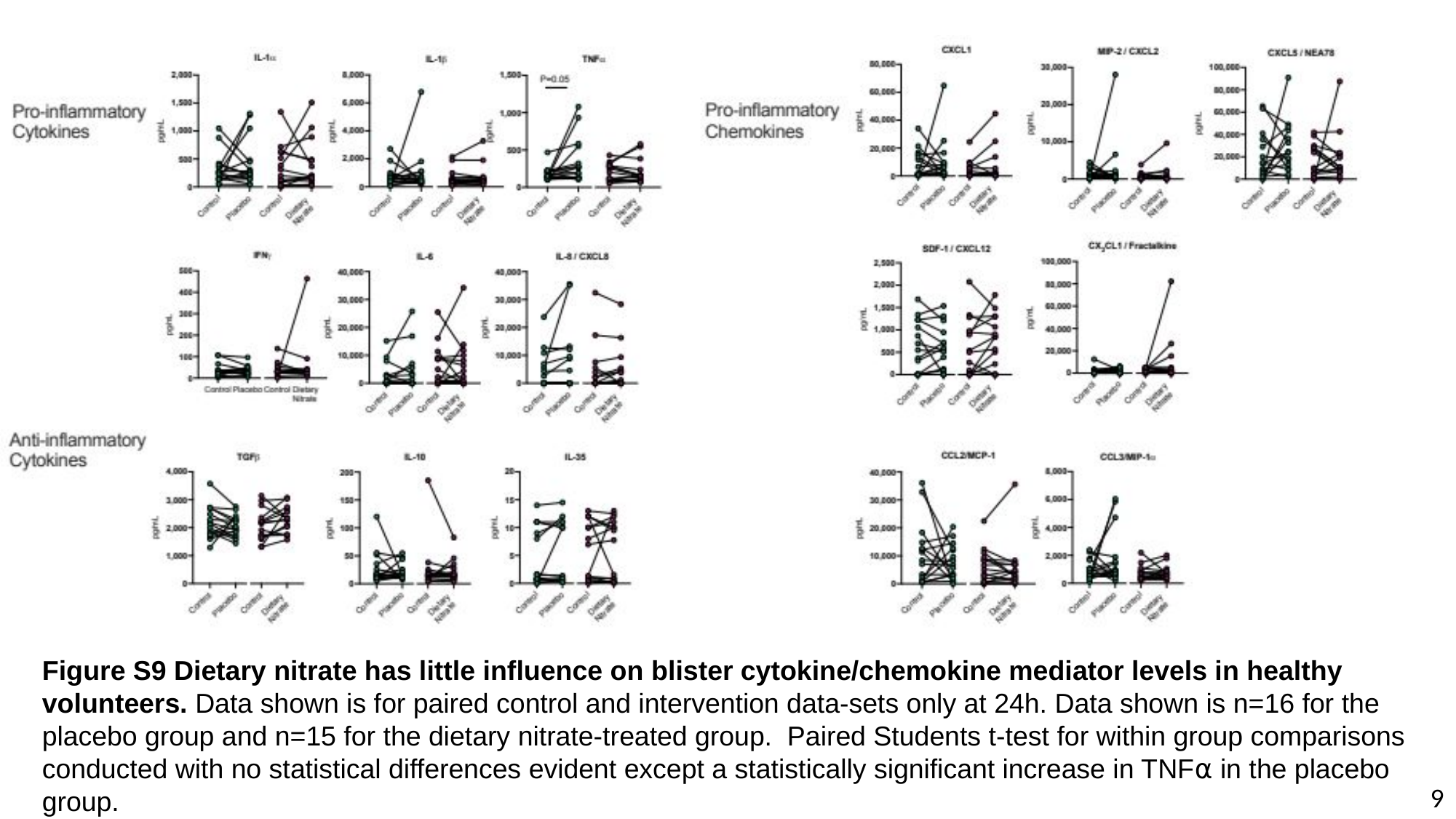

Figure S9 Dietary nitrate has little influence on blister cytokine/chemokine mediator levels in healthy volunteers. Data shown is for paired control and intervention data-sets only at 24h. Data shown is n=16 for the placebo group and n=15 for the dietary nitrate-treated group. Paired Students t-test for within group comparisons conducted with no statistical differences evident except a statistically significant increase in TNF⍺ in the placebo group.
9

## Slide 11
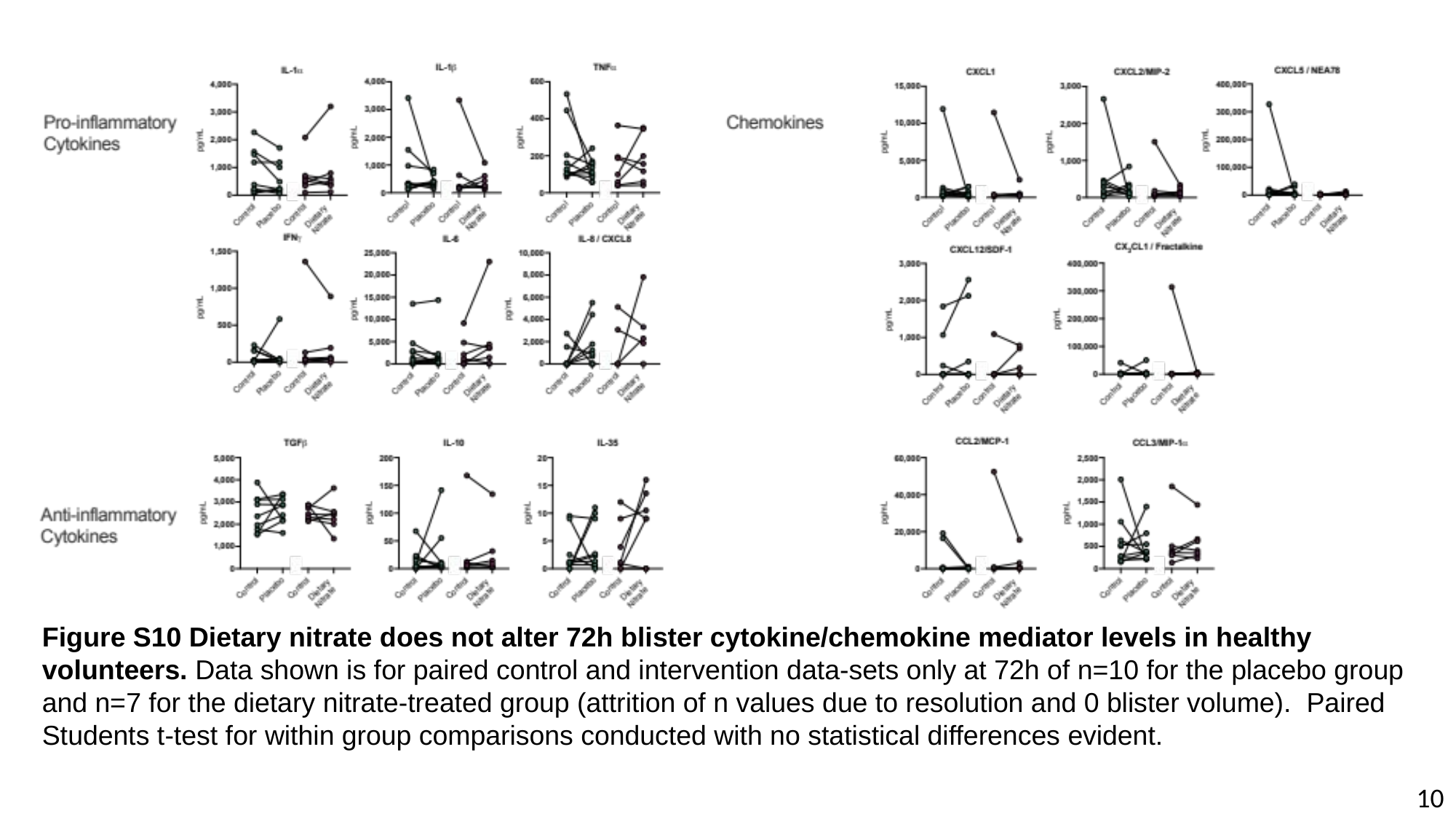

Figure S10 Dietary nitrate does not alter 72h blister cytokine/chemokine mediator levels in healthy volunteers. Data shown is for paired control and intervention data-sets only at 72h of n=10 for the placebo group and n=7 for the dietary nitrate-treated group (attrition of n values due to resolution and 0 blister volume). Paired Students t-test for within group comparisons conducted with no statistical differences evident.
10

## Slide 12
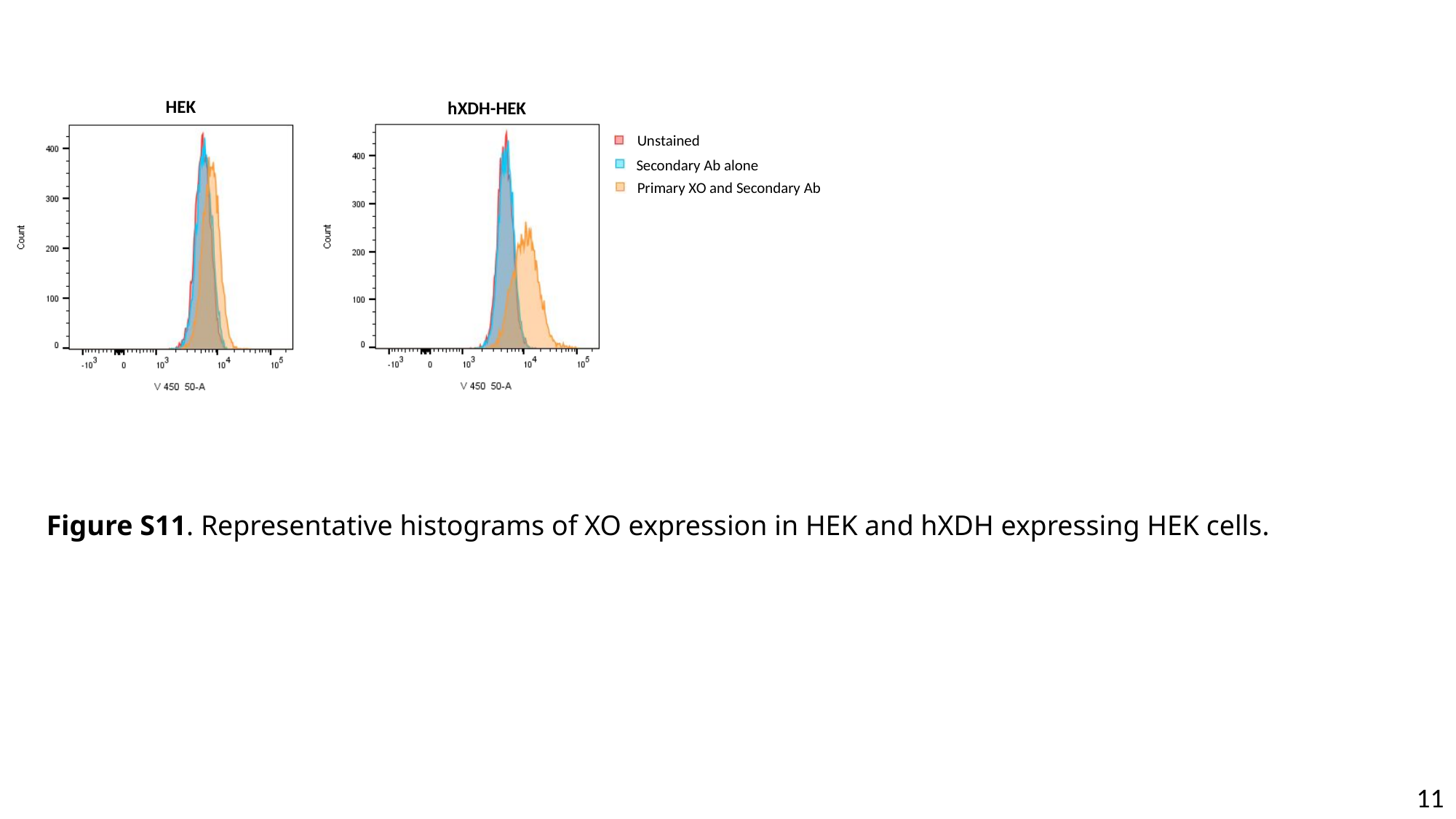

HEK
hXDH-HEK
Unstained
Secondary Ab alone
Primary XO and Secondary Ab
Figure S11. Representative histograms of XO expression in HEK and hXDH expressing HEK cells.
11

## Slide 13
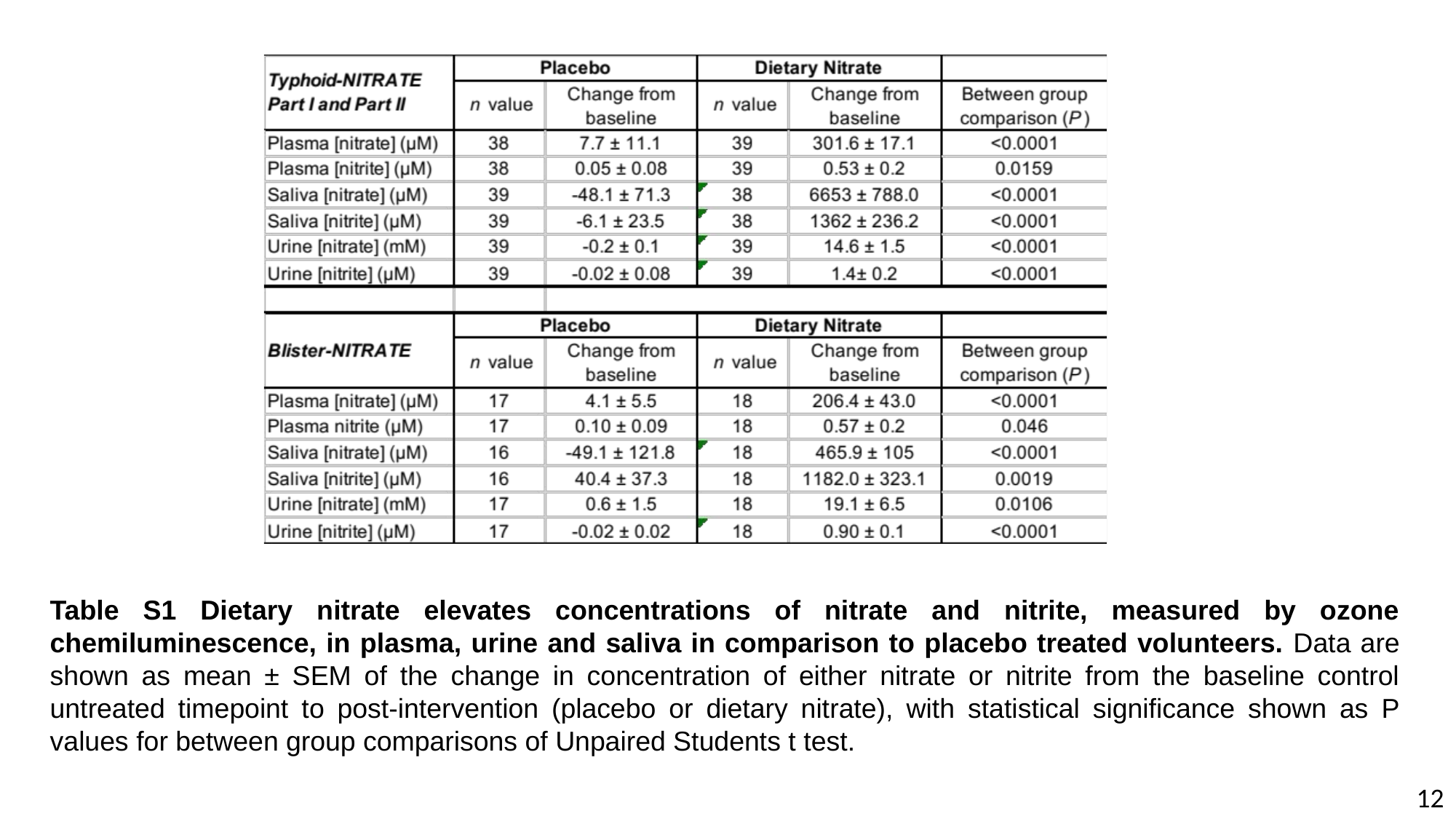

Table S1 Dietary nitrate elevates concentrations of nitrate and nitrite, measured by ozone chemiluminescence, in plasma, urine and saliva in comparison to placebo treated volunteers. Data are shown as mean ± SEM of the change in concentration of either nitrate or nitrite from the baseline control untreated timepoint to post-intervention (placebo or dietary nitrate), with statistical significance shown as P values for between group comparisons of Unpaired Students t test.
12

## Slide 14
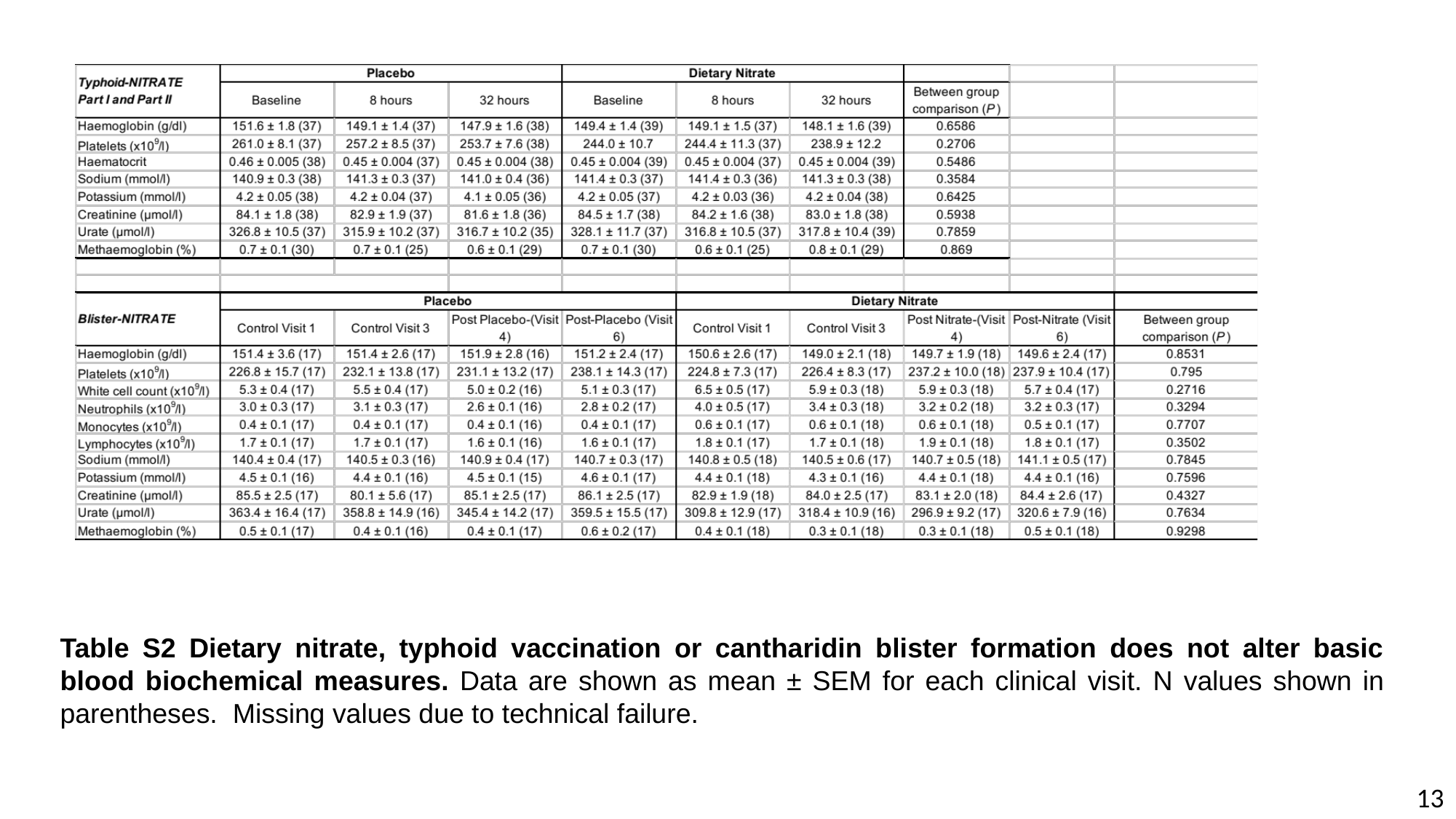

Table S2 Dietary nitrate, typhoid vaccination or cantharidin blister formation does not alter basic blood biochemical measures. Data are shown as mean ± SEM for each clinical visit. N values shown in parentheses. Missing values due to technical failure.
13

## Slide 15
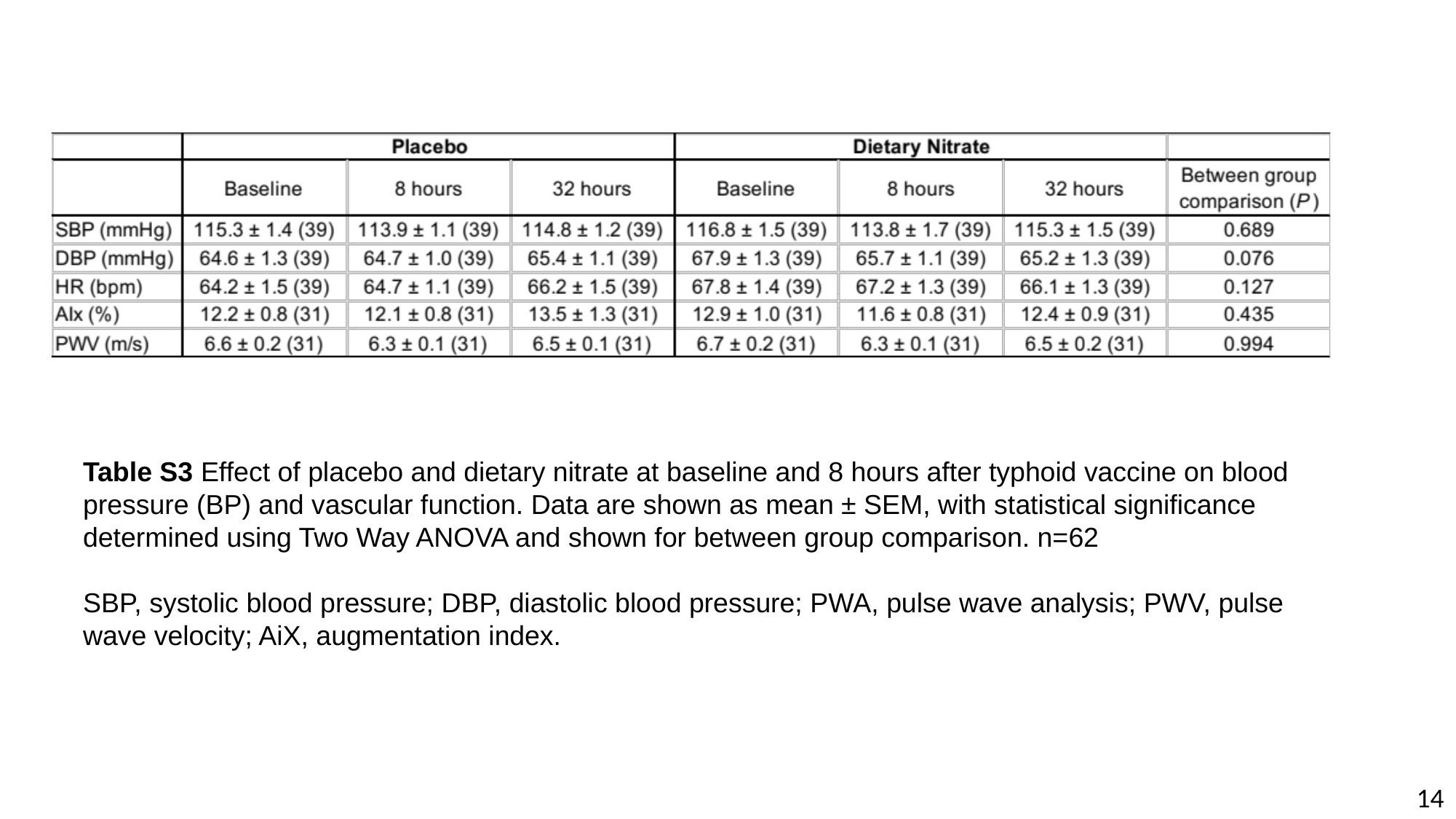

Table S3 Effect of placebo and dietary nitrate at baseline and 8 hours after typhoid vaccine on blood pressure (BP) and vascular function. Data are shown as mean ± SEM, with statistical significance determined using Two Way ANOVA and shown for between group comparison. n=62
SBP, systolic blood pressure; DBP, diastolic blood pressure; PWA, pulse wave analysis; PWV, pulse wave velocity; AiX, augmentation index.
14

## Slide 16
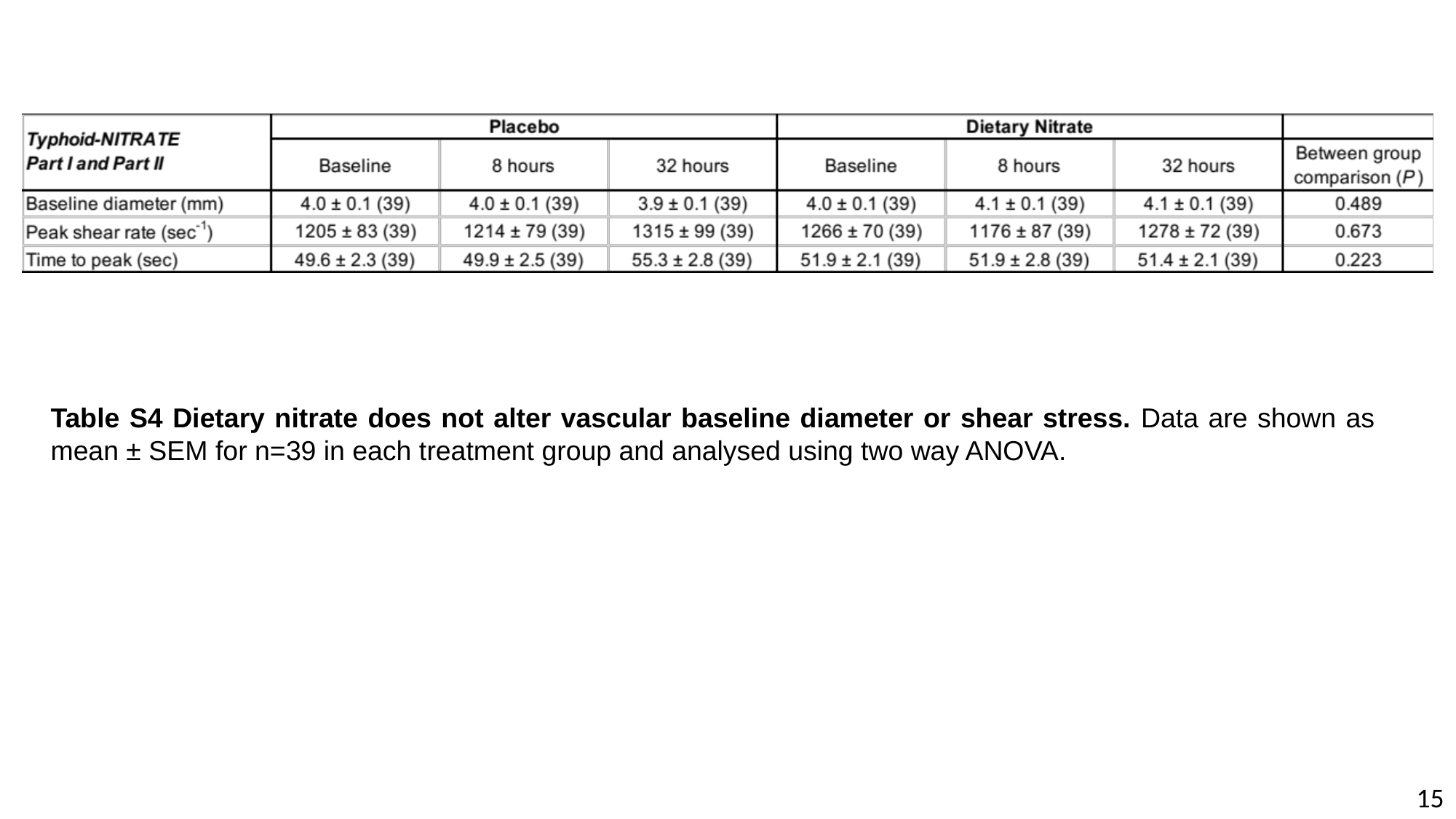

Table S4 Dietary nitrate does not alter vascular baseline diameter or shear stress. Data are shown as mean ± SEM for n=39 in each treatment group and analysed using two way ANOVA.
15
